# Supplementary material for: Use of suboptimal control arms in randomized clinical trials of investigational cancer drugs in China, 2016–2021: An observational study
Source: PLoS Med. 2023 Dec 12;20(12):e1004319. doi: 10.1371/journal.pmed.1004319 (PMC10715645; doi:10.1371/journal.pmed.1004319)
Supplement: S1 Text — Box A. Key words and research strategy to identify clinical practice guidelines other than Chinese Society of Clinical Oncology. Box B. Key words and research strategy to identify oncology trials. Table A. Characteristics of oncology guidelines used to evaluate the quality of trial comparator. Table B. Randomized oncology clinical trials with a potentially optimal control arm in China, 2016–2021. Table C. Randomized oncology clinical trials with a suboptimal control arm in China, 2016–2021. Table D. Primary endpoints used in sample randomized oncology trials with published results. Table E. Primary endpoint results and statistical significance of sample randomized oncology trials. Fig A. Use of suboptimal control arm in randomized controlled oncology trials in China, by applicable guideline type. Table F. Guideline published duration before the ethical approval of sample randomized controlled trials. (DOCX) [file pmed.1004319.s002.docx]

**Use of Suboptimal Control Arms in Randomized Clinical Trials of Investigational Cancer Drugs in China, 2016-2021: An Observational Study**

Yichen Zhang; Dingyi Chen; Siyuan Cheng; Zhizhou Liang; Lu Yang; Qian Li; Lin Bai; Huangqianyu Li; Wei Liu; Luwen Shi; Xiaodong Guan

**Web-only Supporting Information**

**Table of contents**

[Box A. Key words and research strategy to identify clinical practice guidelines other than Chinese Society of Clinical Oncology 1](#_Toc150985224)

[Box B. Key words and research strategy to identify oncology trials 1](#_Toc150985225)

[Table A. Characteristics of oncology guidelines used to evaluate the quality of trial comparator 2](#_Toc150985226)

[Table B. Randomized oncology clinical trials with a potentially optimal control arm in China, 2016-2021 6](#_Toc150985227)

[Table C. Randomized oncology clinical trials with a suboptimal control arm in China, 2016-2021 10](#_Toc150985228)

[Table D. Primary endpoints used in sample randomized oncology trials with published results 19](#_Toc150985229)

[Table E. Primary endpoint results and statistical significance of sample randomized oncology trials 20](#_Toc150985230)

[Fig A. Use of suboptimal control arm in randomized controlled oncology trials in China, by applicable guideline type 25](#_Toc150985231)

[Table F. Guideline published duration before the ethical approval of sample randomized controlled trials 26](#_Toc150985232)

## Box A. Key words and research strategy to identify clinical practice guidelines other than Chinese Society of Clinical Oncology

| **Databases**  China National Knowledge Infrastructure  WanFang Database  **Key Chinese search terms to identify clinical practical guidelines**  #1 “指南 (guideline) ”  #2 “癌 (neoplasm, cancer, carcinoma) ” or “瘤 (tumor, lymphoma, sarcoma, melanoma) ” or “白血病 (leukemia) ”  **Research strategies**  #1 AND #2  Time: from database inception to December 2021  The search language was limited to Chinese. |
| --- |

## Box B. Key words and research strategy to identify oncology trials

| **Database**  China’s National Medical Products Administration Drug Clinical Trial Registration and Information Disclosure Platform  **Key Chinese search terms to identify oncology trials**  #1 “癌 (neoplasm, cancer, carcinoma)”  #2 “瘤 (tumor, lymphoma, sarcoma, melanoma)”  #3 “白血病 (leukemia)”  **Research strategies**  1#or #2 or #3  the time limit was from inception to December 2021. |
| --- |

## Table A. Characteristics of oncology guidelines used to evaluate the quality of trial comparator

| **No.** | **Guideline Issue Time** | **Guideline** | **Academic Society** | **No. Trial (%, n=453)** |
| --- | --- | --- | --- | --- |
| **Biliary Tract** | |  |  |  |
| 1 | Nov-15 | Guideline for Diagnosis and Treatment of Gallbladder Carcinoma (2015 Edition) | Biliary Surgery Group of Surgery Branch, Chinese Medical Association | 5 (1.1) |
| 2 | May-20 | 2020Guideline of CSCO-Biliary Tract Cancer | CSCO | 3 (0.7) |
| **Breast** | |  |  |  |
| 3 | Sep-15 | Guideline for Diagnosis and Treatment of Breast Cancer (2015 Edition) | Chinese Anti-Cancer Association, Committee of Breast Cancer Society | 7 (1.5) |
| 4 | Apr-17 | 2017Guideline of CSCO-Breast Cancer | CSCO | 5 (1.1) |
| 5 | Apr-18 | 2018Guideline of CSCO-Breast Cancer | CSCO | 11 (2.4) |
| 6 | Apr-19 | 2019Guideline of CSCO-Breast Cancer | CSCO | 15 (3.3) |
| 7 | May-20 | 2020Guideline of CSCO-Breast Cancer | CSCO | 10 (2.2) |
| 8 | Apr-21 | 2021Guideline of CSCO-Breast Cancer | CSCO | 20 (4.4) |
| **Cervix** | |  |  |  |
| 9 | Jun-18 | Guideline for Diagnosis and Treatment of Cervical Cancer (4th Edition) | Committee of Gynecological Oncology, Chinese Anti-Cancer Association | 4 (0.9) |
| 10 | Jun-21 | Guideline for Diagnosis and Treatment of Cervical Cancer (2021 Edition) | Committee of Gynecological Oncology, Chinese Anti-Cancer Association | 3 (0.7) |
| **Colon and Rectum** | | |  |  |
| 11 | Jul-13 | Standardization of Diagnosis and Treatment for Colorectal Cancer (2013 Edition) | National Health Commission of the People's Republic of China | 1 (0.2) |
| 12 | Aug-16 | Guideline for Diagnosis and Comprehensive Treatment of Colorectal Cancer with Liver Metastases (2016 Edition) | Section of Gastrointestinal Surgery, Branch of Surgery, Chinese Medical Association | 1 (0.2) |
| 13 | Apr-17 | 2017Guideline of CSCO-Colorectal Cancer | CSCO | 2 (0.4) |
| 14 | Apr-19 | 2019Guideline of CSCO-Colorectal Cancer | CSCO | 1 (0.2) |
| 15 | Apr-20 | 2020Guideline of CSCO-Colorectal Cancer | CSCO | 1 (0.2) |
| 16 | Apr-21 | 2021Guideline of CSCO-Colorectal Cancer | CSCO | 2 (0.4) |
| **Endometrium** | |  |  |  |
| 17 | Aug-18 | Guideline for Diagnosis and Management of Endometrial Cancer (2018 Edition) | Committee of Gynecological Oncology, Chinese Anti-Cancer Association | 2 (0.4) |
| 18 | Jun-21 | Guideline for Diagnosis and Management of Endometrial Cancer (2021 Edition) | Committee of Gynecological Oncology, Chinese Anti-Cancer Association | 2 (0.4) |
| **Glioma** | |  |  |  |
| 19 | Feb-16 | Guideline for Diagnosis and Treatment of Central Nervous System Glioma (2015 Edition) | Guideline Editorial Committee | 3 (0.7) |
| **Head and Neck** | |  |  |  |
| 20 | Apr-18 | 2018Guideline of CSCO-Head and Neck Cancer | CSCO | 2 (0.4) |
| 21 | Apr-19 | 2019Guideline of CSCO-Head and Neck Cancer | CSCO | 3 (0.7) |
| 22 | Jun-20 | 2020Guideline of CSCO-Head and Neck Cancer | CSCO | 1 (0.2) |
| 23 | Jun-20 | 2020Guideline of CSCO-Nasopharynx Cancer | CSCO | 3 (0.7) |
| 24 | Apr-21 | 2021Guideline of CSCO-Head and Neck Cancer | CSCO | 2 (0.4) |
| **Kidney** | |  |  |  |
| 25 | Sep-15 | Guideline for Diagnosis and Treatment of Kidney Cancer (2015 Edition) | Committee of Kidney Cancer, Chinses Society of Clinical Oncology | 2 (0.4) |
| 26 | Apr-19 | 2019Guideline of CSCO-Kidney Cancer | CSCO | 3 (0.7) |
| **Leukemia** | |  |  |  |
| 27 | Aug-16 | Guideline for Diagnosis and Treatment of Chronic Myelogenous Leukemia in China (2016 Edition) | Chinese Society of Hematology, Chinses Medical Association | 1 (0.2) |
| 28 | Oct-16 | Guideline for Diagnosis and Treatment of Acute Lymphoblastic Leukemia in China (2016 Edition) | Chinese Society of Hematology, Chinses Medical Association | 1 (0.2) |
| 29 | Mar-17 | Guideline for Diagnosis and Treatment of Adult Acute Myelogenous Leukemia in China (relapse/refractory) (2017 Edition) | Chinese Hematology Association | 4 (0.9) |
| 30 | Mar-17 | Guideline for Diagnosis and Treatment of Adult Acute Myeloid Leukemia in China (not Acute Promyelocytic Leukemia) (2017 Edition) | Chinese Society of Hematology, Chinses Medical Association | 6 (1.3) |
| 31 | Feb-19 | Guideline for Diagnosis and Treatment of Myelodysplastic Syndromes (2019 Edition) | Chinese Society of Hematology, Chinses Medical Association | 2 (0.4) |
| 32 | Apr-21 | 2021Guideline of CSCO-Hematological Malignancies | CSCO | 1 (0.2) |
| **Liver** | |  |  |  |
| 33 | Jun-17 | Standardization of Diagnosis and Treatment for Hepatocellular Carcinoma (2017 Edition) | National Health Commission of the People's Republic of China | 3 (0.7) |
| 34 | Aug-18 | 2018Guideline of CSCO-Hepatocellular Carcinoma | CSCO | 19 (4.2) |
| 35 | Jul-20 | 2020Guideline of CSCO-Hepatocellular Carcinoma | CSCO | 11 (2.4) |
| **Lung** | |  |  |  |
| 36 | Oct-15 | Guideline for Diagnosis and Management of ALK mutation positive NSCLC (2015 Edition) | Committee of Tumor Biomarker, Chinses Society of Clinical Oncology | 1 (0.2) |
| 37 | Apr-16 | 2016Guideline of CSCO-Lung Cancer | CSCO | 30 (6.6) |
| 38 | Jul-18 | 2018Guideline of CSCO-Lung Cancer | CSCO | 30 (6.6) |
| 39 | Apr-19 | 2019Guideline of CSCO-Lung Cancer | CSCO | 33 (7.3) |
| 40 | May-20 | 2020Guideline of CSCO-Non-Small Cell Lung Cancer | CSCO | 33 (7.3) |
| 41 | May-20 | 2020Guideline of CSCO-Small Cell Lung Cancer | CSCO | 4 (0.9) |
| 42 | Sep-21 | 2021Guideline of CSCO-Non-Small Cell Lung Cancer | CSCO | 9 (2.0) |
| 43 | Sep-21 | 2021Guideline of CSCO-Small Cell Lung Cancer | CSCO | 3 (0.7) |
| **Lymphoma** | |  |  |  |
| 44 | Sep-13 | Guideline for Diagnosis and Treatment of Diffuse Large B Cell Lymphoma (2013 Edition) | Chinese Society of Hematology, Chinses Medical Association | 1 (0.2) |
| 45 | Sep-18 | 2018Guideline of CSCO-Lymphoma | CSCO | 3 (0.7) |
| 46 | Apr-19 | 2019Guideline of CSCO-Lymphoma | CSCO | 8 (1.8) |
| 47 | Feb-20 | 2020Guideline of CSCO-Lymphoma | CSCO | 4 (0.9) |
| 48 | Apr-21 | 2021Guideline of CSCO-Lymphoma | CSCO | 8 (1.8) |
| **Melanoma** | |  |  |  |
| 49 | Apr-17 | 2017Guideline of CSCO-Melanoma | CSCO | 1 (0.2) |
| 50 | Apr-19 | 2019Guideline of CSCO-Melanoma | CSCO | 2 (0.4) |
| **Multiple Myeloma** | | |  |  |
| 51 | Dec-15 | Guideline for Diagnosis and Management of Multiple Myeloma in China (2015 Revision) | Chinese Society of Hematology, Chinses Medical Association | 3 (0.7) |
| 52 | Nov-17 | Guideline for Diagnosis and Management of Multiple Myeloma in China (2017 Revision) | Chinese Society of Hematology, Chinses Medical Association | 8 (1.8) |
| 53 | May-20 | Guideline for Diagnosis and Management of Multiple Myeloma in China (2020 Revision) | Chinese Hematology Association | 1 (0.2) |
| 54 | Apr-21 | 2021Guideline of CSCO-Hematological Malignancies | CSCO | 1 (0.2) |
| **Neuroendocrine Carcinoma** | | |  |  |
| 55 | Aug-21 | 2021Guideline of CSCO-Neuroendocrine Cancer | CSCO | 1 (0.2) |
| **Oesophagus** | |  |  |  |
| 56 | Sep-17 | Guide of Rational Use of Drug on Malignant Tumors in Digestive Tract | Committee of Experts on Rational Drug Use National Health and Family Planning Commission of the People's Republic of China | 9 (2.0) |
| 57 | Apr-19 | 2019Guideline of CSCO-Esophageal Cancer | CSCO | 4 (0.9) |
| 58 | May-20 | 2020Guideline of CSCO-Esophageal Cancer | CSCO | 4 (0.9) |
| 59 | Apr-21 | 2021Guideline of CSCO-Esophageal Cancer | CSCO | 1 (0.2) |
| **Ovary** | |  |  |  |
| 60 | Jul-18 | Guideline for Diagnosis and Treatment of Malignant Ovary Tumors (4th Edition) | Committee of Gynecological Oncology, Chinese Anti-Cancer Association | 1 (0.2) |
| 61 | Apr-19 | 2019Guideline of CSCO-Ovarian Cancer | CSCO | 4 (0.9) |
| 62 | Aug-20 | 2020Guideline of CSCO-Ovarian Cancer | CSCO | 4 (0.9) |
| 63 | Aug-21 | 2021Guideline of CSCO-Ovarian cancer | CSCO | 3 (0.7) |
| **Pancreas** | |  |  |  |
| 64 | Nov-14 | Guideline for Diagnosis and Treatment of Pancreatic Cancer (2014 Edition) | Chinese Pancreatic Surgery Association, Chinese Society of Surgery, Chinese Medical Association | 1 (0.2) |
| 65 | Apr-18 | 2018Guideline of CSCO-Pancreatic Cancer | CSCO | 1 (0.2) |
| 66 | Apr-19 | 2019Guideline of CSCO-Pancreatic Cancer | CSCO | 1 (0.2) |
| 67 | Jun-20 | 2020Guideline of CSCO-Pancreatic Cancer | CSCO | 2 (0.4) |
| **Prostate** | |  |  |  |
| 68 | Aug-14 | Chinese Urological Disease Diagnosis and Treatment Guideline Manual (2014 edition) | Chinese Society of Urology, Chinese Medical Association | 10 (2.2) |
| 69 | Dec-18 | Standardization of Diagnosis and Treatment for Prostate Cancer (2018 Edition) | National Health Commission of the People's Republic of China | 8 (1.8) |
| 70 | Aug-20 | 2020Guideline of CSCO-Prostatic Cancer | CSCO | 5 (1.1) |
| 71 | Aug-21 | 2021Guideline of CSCO-Prostatic Cancer | CSCO | 2 (0.4) |
| **Soft Tissue Sarcoma** | | |  |  |
| 72 | Apr-21 | 2021Guideline of CSCO-Soft Tissue Sarcoma | CSCO | 1 (0.2) |
| **Stomach** | |  |  |  |
| 73 | Apr-13 | Standardization of Diagnosis and Treatment for Gastric Cancer (2013 Edition) | National Health Commission of the People's Republic of China | 3 (0.7) |
| 74 | Apr-17 | 2017Guideline of CSCO-Stomach Cancer | CSCO | 3 (0.7) |
| 75 | Apr-18 | 2018Guideline of CSCO-Stomach Cancer | CSCO | 7 (1.5) |
| 76 | Apr-19 | 2019Guideline of CSCO-Stomach Cancer | CSCO | 9 (2.0) |
| 77 | Sep-20 | 2020Guideline of CSCO-Stomach Cancer | CSCO | 2 (0.4) |
| 78 | Apr-21 | 2021Guideline of CSCO-Stomach Cancer | CSCO | 10 (2.2) |
| **Thyroid** | |  |  |  |
| 79 | Sep-14 | Guideline for 131I Therapy of Differentiated Thyroid Cancer | Chinese Society of Nuclear Medicine, Chinses Medical Association | 2 (0.4) |
| 80 | Apr-19 | 2019Guideline of CSCO-Thyroid Cancer | CSCO | 1 (0.2) |
| 81 | Aug-21 | 2021Guideline of CSCO-Thyroid Cancer | CSCO | 1 (0.2) |
| **Urothelium** | |  |  |  |
| 82 | Dec-18 | Standardization of Diagnosis and Treatment for Bladder Cancer (2018 Edition) | National Health Commission of the People's Republic of China | 2 (0.4) |
| 83 | Sep-20 | 2020Guideline of CSCO-Urothelial Cancer | CSCO | 4 (0.9) |
| 84 | Aug-21 | 2021Guideline of CSCO-Urothelial Cancer | CSCO | 2 (0.4) |

***Abbreviations:*** *CSCO, Chinese Society of Clinical Oncology.*

## Table B. Randomized oncology clinical trials with a potentially optimal control arm in China, 2016-2021

| **No.** | **CTR** | **Phase** | **Enrolment Region** | **Cancer Site** | **Stage** | **Target** | **Line of Therapy** | **Investigational Regimen** | **Combination** | **Control** | **Chinese Guideline Recommended Regimen** | **Potentially optimal reason** |
| --- | --- | --- | --- | --- | --- | --- | --- | --- | --- | --- | --- | --- |
| 1 | CTR20202323 | II/III | International multicenter | Head and Neck | Recurrent or metastatic | / | 1st-line | GSK3359609 | Pembrolizumab+ 5-FU-Platinum-Based Chemotherapy | Placebo+ Pembrolizumab+ 5FU-Platinum-Based Chemotherapy | Cisplatin/Carboplatin+ 5-FU+ Cetuximab OR Cisplatin + Docetaxel+ Cetuximab OR Cisplatin/Carboplatin+ Paclitaxel± Cetuximab | FDA approved therapy |
| 2 | CTR20201774 | III | International multicenter | Head and Neck | Recurrent or metastatic | PD-L1+ | 1st-line | Pembrolizumab | Lenvatinib | Pembrolizumab+ Placebo | Cisplatin/Carboplatin+ 5-FU OR Cisplatin+ Paclitaxel OR Cisplatin+ Docetaxel | FDA approved therapy |
| 3 | CTR20201147 | II/III | International multicenter | Head and Neck | Recurrent or metastatic | PD-L1+ | 1st-line | GSK3359609 | Pembrolizumab | Pembrolizumab+ Placebo | Cisplatin/Carboplatin+ 5-FU OR Cisplatin+ Paclitaxel OR Cisplatin+ Docetaxel | FDA approved therapy |
| 4 | CTR20182342 | III | International multicenter | Lung | Stage IIIB/IIIC/IV | ALK+ | 2nd-line | Brigatinib | / | Alectinib | Crizotinib | FDA approved therapy |
| 5 | CTR20211590 | III | International multicenter | Stomach | Metastatic and/or unresectable | HER2+ | 2nd-line | Trastuzumab deruxtecan | / | Ramucirumab+ Paclitaxel | Paclitaxel/Docetaxel/Irinotecan | FDA approved therapy |
| 6 | CTR20170690 | III | International multicenter | Prostate | Metastatic castration resistance | / | 3rd-line | Atezolizumab | enzalutamide | Enzalutamide | Palliative care | FDA approved therapy |
| 7 | CTR20192167 | III | China-only | Lung | Resectable stage IIB-IIIA | / | neoadjuvant+ adjuvant | Serplulimab | Chemotherapy | Placebo+ Chemotherapy | Platinum-Doublet Adjuvant Chemotherapy | FDA approved therapy |
| 8 | CTR20190907 | III | International multicenter | Lung | Unresectable stage IIIB/IIIC/IV | EGFR-, ALK-, ROS1- | 1st-line | Serplulimab | Carboplatin+ Paclitaxel (Albumin Bound) | Placebo+ Paclitaxel (Albumin Bound)+ Carboplatin | Grade of Recommendation I: Chemoradiotherapy OR Platinum+ Gemcitabine (Level of Evidence: 1A) /Docetaxel (1A)/Paclitaxel (1A)/Paclitaxel Liposome (2A) OR Gemcitabine+ Docetaxel/Vinorelbine (1) | FDA approved therapy |
| 9 | CTR20212256 | III | International multicenter | Lung | Locally advanced or metastatic | PD-L1+ | 1st-line | Atezolizumab | Tiragolumab | Placebo+ Atezolizumab | Grade of Recommendation I: Pemetrexed+ Platinum+ Pemetrexed Maintenance (Level of Evidence: 1A) OR Bevacizumab+ Platinum-Doublet Chemotherapy+ Bevacizumab maintenance (1A&2A) OR Cisplatin/Carboplatin+ Gemcitabine (1A)/Docetaxel (1A) OR Paclitaxel/Paclitaxel Lipdosome (1A/2A)/Vinorelbine(1A)/Pemetrexed(1A) OR Gemcitabine+ Docetaxel (1) OR Gemcitabine+ Vinorelbine(1) OR Pembrolizumab(PD-L1 TPS ≥ 50% (1A), PD-L1 TPS 1~49%(2A)) OR Pembrolizumab+ Pemetrexed+ Platinum (1A) | FDA approved therapy |
| 10 | CTR20200368 | III | International multicenter | Melanoma | Stage III/IV | / | 1st-line | Pembrolizumab | Lenvatinib | Pembrolizumab+ Placebo | Grade of Recommendation I: Skin/Acral Melanoma: PD-1 Targeting Monoclonal Antibodies; Mucosal Melanoma: Chemotherapy + antiangiogenic drugs | FDA approved therapy |
| 11 | CTR20190378 | III | International multicenter | Breast | Locally advanced or metastatic | HER2+ | 2nd-line | DS8201a | / | Ado-Trastuzumab-Emtansine | Grade of Recommendation I: Lapatinib+ Capecitabine | Global standard of care |
| 12 | CTR20211008 | III | International multicenter | Breast | Locally advanced or metastatic | HER2+, PD-L1+ | 2nd-line | Atezolizumab | Trastuzumab Emtansine | Trastuzumab Emtansine+ Placebo | Grade of Recommendation I: Pyrotinib+ Capecitabine | Global standard of care |
| 13 | CTR20212619 | III | International multicenter | Breast | Locally advanced or metastatic | HER2+ | 2nd-line | Tucatinib | Trastuzumab Emtansine | Placebo+ Trastuzumab Emtansine | Grade of Recommendation I: Pyrotinib+ Capecitabine | Global standard of care |
| 14 | CTR20180927 | III | International multicenter | Liver | / | / | adjuvant | Nivolumab | / | Placebo | TACE | Global standard of care (no consensual adjuvant therapy for hepatocellalar carcinoma) |
| 15 | CTR20190736 | III | International multicenter | Liver | / | / | adjuvant | Durvalumab | Placebo/Bevacizumab | Placebo | TACE | Global standard of care (no consensual adjuvant therapy for hepatocellalar carcinoma) |
| 16 | CTR20192580 | III | International multicenter | Liver | / | / | adjuvant | Atezolizumab | Bevacizumab | Active Monitoring | TACE | Global standard of care (no consensual adjuvant therapy for hepatocellalar carcinoma) |
| 17 | CTR20181920 | III | International multicenter | Leukemia | / | IDH1+ | 1st-line | Ivosidenib | Azacitidine | Placebo+ Azacitidine | Induction Remission: Standard-Dose Cytarabine+ Idarubicin/Daunorubicin | Recommended in later guideline |
| 18 | CTR20191971 | III | International multicenter | Leukemia | / | / | 1st-line | Pracinostat | Azacitidine | Placebo+ Azacitidine | Induction Remission: Standard-Dose Cytarabine+ Idarubicin/Daunorubicin | Recommended in later guideline |
| 19 | CTR20192195 | III | International multicenter | Prostate | Metastatic | DNA repair gene defects positive or negative | 1st-line | Niraparib | Abiraterone+ Prednisone | Placebo+ Abiraterone+ Prednisone | Docetaxel+ ADT | Recommended in later guideline |
| 20 | CTR20191830 | III | International multicenter | Stomach | Stage III (T≥3, N≥1, M0) | PD-L1+ | neoadjuvant+ adjuvant | Serplulimab | Oxaliplatin+ Fluorouracil/Capecitabine+ Calcium Folinate | Placebo+ Oxaliplatin+ Fluorouracil/Capecitabine+ Calcium Folinate | Neoadjuvant Therapy: Chemoradiotherapy OR FLOT4; Adjuvant Therapy: XELOX | Recommended in later guideline |
| 21 | CTR20171498 | III | International multicenter | Leukemia | Not suitable for standard induction therapy | / | 1st-line | ABT-199 | Azacitidine | Placebo+ Azacitidine | Low-intensity chemotherapy: Decitabine± low-dose chemotherapy OR low-dose chemotherapy±G-CSF OR low-dose Cytarabine; Supportive care | Recommended in later guideline |
| 22 | CTR20181749 | III | International multicenter | Leukemia | Not suitable for standard induction therapy | / | 1st-line | Glasdegib | Azacitidine OR Cytarabine+ Daunorubicin | Placebo+ Azacitidine OR Placebo+ Cytarabine+ Daunorubicin | Low-intensity chemotherapy: Decitabine± low-dose chemotherapy OR low-dose chemotherapy±G-CSF OR low-dose Cytarabine; Supportive care | Recommended in later guideline |
| 23 | CTR20182551 | III | International multicenter | Stomach | Locally advanced or metastatic | HER2+ | 1st-line | Pembrolizumab | Trastuzumab+ Fluorouracil/Capecitabine+ Cisplatin/Oxaliplatin | Placebo+ Trastuzumab+ Fluorouracil/Capecitabine+ Cisplatin/Oxaliplatin | Grade of Recommendation I: Trastuzumab+ Fluorouracil/Capecitabine+ Cisplatin | Recommended in later guideline |
| 24 | CTR20200660 | II/III | International multicenter | Stomach | Locally advanced or metastatic | HER2+, PD-L1+ | 1st-line | Margetuximab | INCMGA00012+ Chemotherapy+ Checkpoint Inhibitor | Trastuzumab+ Oxaliplatin+ Fluorouracil/Capecitabine+ Calcium Folinate | Grade of Recommendation I: Trastuzumab+ Fluorouracil/Capecitabine+ Cisplatin | Recommended in later guideline |

***Abbreviations:***

*5-FU, 5-fluorouracil; ADT, androgen deprivation therapy; ALK, anaplastic lymphoma kinase; DNA, deoxyribonucleic acid; EGFR, epidermal growth factor receptor; FDA, the United States Food and Drug Administration; FLOT, fluorouracil plus leucovorin, oxaliplatin and docetaxel; G-CSF, granulocyte colony-stimulating factor; HER2, human epidermal growth factor receptor 2; IDH1, isocitrate dehydrogenase 1; PD-L1, programmed cell death-ligand 1; ROS1, recombinant c-ros oncogene 1; TACE, transarterial chemoembolization; TPS, tumor cell proportion score; XELOX, capecitabine and oxaliplatin.*

## Table C. Randomized oncology clinical trials with a suboptimal control arm in China, 2016-2021

| **No.** | **CTR** | **IRB Approval Time** | **Phase** | **Enrolment Region** | **Cancer Site** | **Stage** | **Target** | **Line of Therapy** | **Investigational Regimen** | **Combination** | **Control** | **Guideline Recommended Regimen** |
| --- | --- | --- | --- | --- | --- | --- | --- | --- | --- | --- | --- | --- |
| **Category 1** | |  |  |  |  |  |  |  |  |  |  |  |
| 1 | CTR20160389 | Jun-16 | III | China-only | Breast | Advanced | HER2+ | 2nd-line | GB221 | Capecitabine | Placebo+ Capecitabine | Lapatinib+ Capecitabine OR Trastuzumab+ Capecitabine OR Trastuzumab+ Lapatinib OR Trastuzumab OR Other chemotherapy |
| 2 | CTR20200467 | Dec-17 | III | China-only | Breast | Locally advanced or metastatic | HR+, HER2- | 2nd-line | SNDX–275 | Exemestane | Placebo+ Exemestane | Fulvestrant |
| 3 | CTR20171559 | Dec-17 | III | International multicenter | Colon and Rectum | Stage Ⅳ | / | 2nd-line | GB201 | FOLFIRI | GB201 | raltitrexed or best supportive care |
| 4 | CTR20182326 | Nov-18 | III | China-only | Liver | Locally advanced, after radical surgery, have high risk of recurrence | / | adjuvant | Toripalimab | / | Placebo | TACE (Guidelines of Chinese Society of Clinical Oncology-Hepatocellular Carcinoma [2018 and 2020 Edition]: For patients with hepatocellular carcinoma who are at risk of early recurrence, adjuvant TACE after resection has survival benefits.) (According to the trial eligibility criteria, there is no contraindication for TACE in the preplanned participants with high risk of recurrence) |
| 5 | CTR20202509 | Nov-20 | III | China-only | Liver | Locally advanced, after radical surgery, have high risk of recurrence | / | adjuvant | Apatinib | Camrelizumab | Active Monitoring | TACE (the same as above) |
| 6 | CTR20212004 | Jun-21 | III | China-only | Liver | Locally advanced, after radical surgery, have high risk of recurrence | / | adjuvant | Toripalimab | / | Placebo | TACE (the same as above) |
| 7 | CTR20211135 | Feb-21 | III | China-only | Lung-NSCLC | Locally advanced or metastatic | EGFR+ | 1st-line | Almonertinib | Platinum-Doublet Chemotherapy | Almonertinib | Gefitinib OR Erlotinib OR Icotinib OR Afatinib OR Dacomitinib OR Osimertinib (Almonertinib has not been approved for 1st-line therapy in China by Feb 2021) |
| 8 | CTR20212944 | Nov-21 | III | China-only | Lung-NSCLC | Advanced or metastatic | EGFR+ | 1st-line | SHR-1701 | Bevacizumab+ Platinum-Doublet Chemotherapy | Placebo+ SHR-1701+ Platinum-Doublet Chemotherapy | Gefitinib OR Erlotinib OR Icotinib OR Afatinib OR Dacomitinib OR Osimertinib |
| 9 | CTR20190968 | May-19 | III | China-only | Lung-NSCLC | Locally advanced or metastatic | EGFR+ | 2nd-line | Sintilimab | ± IBI305+ Pemetrexed+ Cisplatin | Placebo+ Pemetrexed+ Cisplatin | EGFR-TKI (oligoprogression or central nervous system progression patient) |
| 10 | CTR20190972 | May-19 | III | China-only | Lung-NSCLC | Locally advanced or metastatic | EGFR+ | 2nd-line | Sintilimab | ± IBI305+ Pemetrexed (+Cisplatin) | Placebo+ Pemetrexed+ Cisplatin | EGFR-TKI (oligoprogression or central nervous system progression patient) |
| 11 | CTR20180929 | May-18 | III | International multicenter | Lung-NSCLC | Stage IB (≥4 cm), stage II-IIIA(N2) | PD-L1+ | adjuvant | Nivolumab | Ipilimumab/Platinum-Doublet Chemotherapy | Platinum+ Pemetrexed/Gemcitabine/Docetaxel/Vinorelbine | Adjuvant chemotherapy was NOT recommended for stage IB-IIA NSCLC |
| 12 | CTR20190012 | Nov-18 | III | International multicenter | Lung-NSCLC | Stage II-IIIA/IIIB (T>5 cm, N2) | / | adjuvant | ACZ885 | / | Placebo | Platinum-Doublet Adjuvant Chemotherapy |
| 13 | CTR20182000 | Mar-19 | III | International multicenter | Lung-NSCLC | Stage IB (≥4 cm)-IIIA (T2-3N0,T1-3N1,T1-3N2,T4 N0-1) | ALK+ | adjuvant | Alectinib | / | Platinum+ Pemetrexed/Gemcitabine | Adjuvant chemotherapy was NOT recommended for stage IB-IIA NSCLC |
| 14 | CTR20202460 | Sep-20 | III | China-only | Lung-NSCLC | Stage II-IIIA/IIIB (N2) | EGFR+ | adjuvant | Almonertinib | / | Placebo | Platinum-Doublet Adjuvant Chemotherapy |
| 15 | CTR20210429 | Feb-21 | III | China-only | Lung-NSCLC | Stage II-IIIA | EGFR+ | adjuvant | Furmonertinib | / | Placebo | Platinum-Doublet Adjuvant Chemotherapy |
| 16 | CTR20200932 | Aug-20 | III | International multicenter | Lung-NSCLC | Resectable II-IIIB | / | neoadjuvant+adjuvant | Durvalumab | Neoadjuvant Durvalumab+ Chemotherapy OR Neoadjuvant Durvalumab+ Chemotherapy, Followed by Adjuvant Durvalumab | Neoadjuvant Placebo+ Chemotherapy OR Neoadjuvant Placebo+ Chemotherapy, Followed by Adjuvant Placebo | Platinum-Doublet Adjuvant Chemotherapy (Adjuvant Placebo is NOT recommended) |
| 17 | CTR20210041 | Jul-21 | III | International multicenter | Lung-SCLC | Extensive stage | / | 1st-line | Atezolizumab | Bevacizumab+ Carboplatin/Cisplatin+ Etoposide | Placebo+ Bevacizumab+ Carboplatin/Cisplatin+ Etoposide | Atezolizumab+ Etoposide+ Carboplatin× 4 cycle-atezolizumab maintenance (Priority, 1A); Etoposide/Irinotecan+ Carboplatin/Cisplatin |
| 18 | CTR20210114 | Oct-21 | III | International multicenter | Lung-SCLC | Limited stage | / | 1st-line | Pembrolizumab | Olaparib+ CCRT (Etoposide+ Platinum) | Pembrolizumab+ CCRT (Etoposide+ Platinum) | Chemotherapy (Etoposide+ Cisplatin/Carboplatin)+ Radiotherapy |
| 19 | CTR20210658 | Sep-21 | III | China-only | Lung-SCLC | Recurrent or disease progression | / | 3rd-line | Chiauranib | / | Placebo | Anlotinib |
| 20 | CTR20190595 | Oct-19 | III | International multicenter | Lymphoma | Chronic lymphocytic leukemia | del(17p) or TP53 mutation negative | 1st-line | Acalabrutinib | Venetoclax (+ obinutuzumab) | Bendamustine+ Cyclophosphamide+ Fludarabine+ Rituximab | Chlorambucil+ Rituximab OR Ibrutinib OR Bendamustine± Rituximab OR Fludarabine+ Cyclophosphamide+ Rituximab |
| 21 | CTR20192186 | Jul-19 | III | China-only | Lymphoma | Relapse or refractory | / | 2nd-line | Polatuzumab vedotin | Bendamustine+ Rituximab | Placebo+ Bendamustine+ Rituximab | R-DHAP OR R-ESHAP OR R-ICE OR R-GDP OR R-DA-EPOCH OR R-GemOx OR R-MINE OR R2 |
| 22 | CTR20210442 | May-21 | III | China-only | Lymphoma | Follicular lymphoma grade 1-3a | CD20+ | 2nd-line | MIL62 | Lenalidomide | Lenalidomide | RCHOP OR RCVP OR Bendamustine+ Rituximab (Not Recommended in Patients Who Have Previously Used Bendamustine) OR Lenalidomide+ Rituximab OR 2nd-line Regimen for DLBCL OR Clinical Trials |
| 23 | CTR20211431 | Apr-21 | III | China-only | Multiple Myeloma | Relapse or refractory | / | 2nd-line | ATG-010 (Selinexor) | Bortezomib+ Dexamethasone | Bortezomib+ Dexamethasone | Ixazomib+ Lenalidomide+ Dexamethasone OR Bortezomib+ Pomalidomide+ Dexamethasone OR Carfilzomib+ Dexamethasone OR Daratumumab+ Bortezomib+ Lenalidomide+ Dexamethasone OR Daratumumab+ Lenalidomide+ Dexamethasone OR Daratumumab+ Bortezomib+ Dexamethasone OR Carfilzomib+ Lenalidomide+ Dexamethasone OR Selinexor+ Dexamethasone OR Bortezomib+ Lenalidomide+ Dexamethasone |
| 24 | CTR20170038 | Dec-16 | III | International multicenter | Multiple Myeloma | Newly diagnosed | / | maintenance | Ixazomib | / | Placebo | Lenalidomide/Thalidomide/Bortezomib+ Thalidomide/Prednisone |
| 25 | CTR20180718 | Jun-18 | III | International multicenter | Multiple Myeloma | Newly diagnosed | / | maintenance | Ixazomib | / | Placebo | Lenalidomide/Bortezomib/Thalidomide± Glucocorticoids |
| 26 | CTR20180183 | Jan-18 | III | International multicenter | Oesophagus | Stage II/III | / | adjuvant | Nivolumab | / | Placebo | XELOX OR XP OR S-1 |
| 27 | CTR20192330 | Sep-19 | III | China-only | Ovary | Stage Ⅲ/Ⅳ, 1st-line therapy did not contain bevacizumab | BRCA+ | 1st-line maintenance | IMP4297 | / | Placebo | Maintenance PARP Inhibitor |
| 28 | CTR20200216 | Oct-19 | III | China-only | Ovary | Stage Ⅲ/Ⅳ | BRCA1/2+ | 1st-line maintenance | Fluzoparib | None(BRCA1/2mutation group)/Apatinib | Placebo | Maintenance PARP Inhibitor |
| 29 | CTR20200217 | Oct-19 | III | China-only | Ovary | Stage Ⅲ/Ⅳ | BRCA1/2+ | 1st-line maintenance | Apatinib | None(BRCA1/2mutation group)/Apatinib | Placebo | Maintenance PARP Inhibitor |
| 30 | CTR20211622 | Jun-21 | III | International multicenter | Ovary | Advanced (FIGO III-IV) | BRCA wild type | 1st-line maintenance | Olaparib | / | Placebo | Niraparib OR Bevacizumab (If 1st-line treatment included Bevacizumab, and the efficacy was evaluated as response) |
| 31 | CTR20190294 | Jan-19 | III | China-only | Ovary | Recurrent | BRCA1/2+ | 2nd-line maintenance | Fluzoparib | / | Placebo | Docetaxel OR Etoposide OR Gemcitabine OR Doxorubicin Liposome± Bevacizumab OR Paclitaxel± Pazopanib OR Paclitaxel+ Bevacizumab OR Topotecan± Bevacizumab OR Bevacizumab OR Olaparib OR Rucaparib |
| 32 | CTR20190132 | May-19 | III | China-only | Prostate | Metastatic hormone-sensitive | / | 1st-line | Enzalutamide | ADT | Placebo+ ADT | Docetaxel+ ADT |
| 33 | CTR20160121 | Jun-16 | III | China-only | Thyroid | Radioiodine-refractory differentiated | / | 2nd-line | E7080 | / | Placebo | Sorafenib |
| 34 | CTR20180191 | Jan-18 | III | China-only | Thyroid | Locally advanced or metastatic | / | 2nd-line | Donafenib | Best supportive care | Placebo+ Best supportive care | Sorafenib |
| 35 | CTR20212032 | Apr-21 | III | International multicenter | Urothelium | High-risk muscle-invasive urothelial carcinoma | ctDNA+ | adjuvant | Atezolizumab | / | Placebo | Adjuvant Chemoradiotherapy |
| **Category 2** | |  |  |  |  |  |  |  |  |  |  |  |
| 36 | CTR20212838 | Sep-21 | II/III | China-only | Stomach | Resectable locally advanced | / | neoadjuvant+adjuvant | SHR-1701 | SOX | Placebo+ SOX | Stage II: XELOX; S-1 |
| 37 | CTR20180332 | Dec-17 | III | China-only | Biliary Tract | Unresectable or metastatic | / | 1st-line | KN035 | Gemcitabine+ Oxaliplatin | Gemcitabine+ Oxaliplatin | Gemcitabine+ Cisplatin |
| 38 | CTR20191887 | Sep-19 | III | International multicenter | Leukemia | Acute lymphoblastic leukemia | Philadelphia chromosome positive | 1st-line | Ponatinib | Low-Dose Chemotherapy | Imatinib+ Low-Dose Chemotherapy | Imatinib/Dasatinib+ Multiple Drug Chemotherapy |
| 39 | CTR20201135 | Mar-20 | III | China-only | Oesophagus | Locally advanced | / | 1st-line | Camrelizumab | CCRT (Paclitaxel+ Cisplatin) | Placebo+ CCRT (Paclitaxel+ Cisplatin) | Paclitaxel+ Carboplatin OR Cisplatin+ 5-FU/Capecitabine OR Vinorelbine+ Cisplatin OR Oxaliplatin+ 5-FU/Capecitabine OR Paclitaxel+ 5-FU/Capecitabine |
| 40 | CTR20212696 | Sep-21 | III | China-only | Soft Tissue Sarcoma | Unresectable locally advanced or metastatic | / | 1st-line | Anlotinib | Epirubicin | Placebo+ Epirubicin | A (Doxorubicin) OR A+ lfosfamide (Epirubicin replacing doxorubicin lacks large-scale clinical evidence) |
| **Category 3** | |  |  |  |  |  |  |  |  |  |  |  |
| 41 | CTR20201708 | Jul-20 | II/III | China-only | Breast | Locally advanced or metastatic | HER2+ | 2nd-line | ARX788 | / | Lapatinib+ Capecitabine | Grade of Recommendation I: Pyrotinib+ Capecitabine; Grade of Recommendation II: Trastuzumab Emtansine OR Lapatinib+ Capecitabine |
| 42 | CTR20212456 | Oct-21 | III | China-only | Biliary Tract | Unresectable, locally advanced, recurrent or metastatic | / | 2nd-line | TQB2450 | Anlotinib | Capecitabine+ Oxaliplatin/Gemcitabine | Grade of Recommendation I: mFOLFOX OR Clinical Trials; Grade of Recommendation II: Irinotecan＋Capecitabine OR FOLFIRI OR Other 1st-line therapy that has not been used (e.g., Capecitabine+ Gemcitabine) OR Regorafenib |
| 43 | CTR20190622 | Jan-19 | III | China-only | Breast | Recurrent or metastatic | HER2+ | 1st-line | Pyrotinib | Trastuzumab+ Docetaxel | Placebo+ Trastuzumab+ Docetaxel | Grade of Recommendation I: Trastuzumab+ (Docetaxel+ Capecitabine)/Vinorelbine; Grade of Recommendation II: Trastuzumab+ Docetaxel± Pertuzumab; Trastuzumab+ Capecitabine |
| 44 | CTR20212139 | Aug-21 | III | China-only | Breast | Locally advanced or metastatic | HR+, HER2- | 1st-line | GB491 (Lerociclib) | Letrozole | Placebo+ Letrozole | Grade of Recommendation I: AI+ CDK4/6 inhibitor OR Fulvestrant; Grade of Recommendation II: AI; Fulvestrant+ CDK4/6 inhibitor |
| 45 | CTR20213286 | Nov-21 | III | China-only | Breast | Locally advanced or metastatic | HR+, HER2- | 1st-line | FCN-437c | Letrozole/Anastrozole± Goserelin | Placebo+ Letrozole/Anastrozole± Goserelin | Grade of Recommendation I: AI+ CDK4/6 inhibitor OR Fulvestrant; Grade of Recommendation II: AI; Fulvestrant+ CDK4/6 inhibitor |
| 46 | CTR20171558 | Jan-18 | III | China-only | Breast | Locally advanced or metastatic | HR+, HER2- | 2nd-line | SNDX–275 | Exemestane | Placebo+ Exemestane | Principal strategy: Fulvestrant; Optional strategy: Steroid AI+ Everolimus; Fulvestrant+ CDK4/6 inhibitor/Everolimus; progestin; AI in another mechanism of action; Tamoxifen OR Toremifene |
| 47 | CTR20211545 | May-21 | III | International multicenter | Breast | Locally advanced or metastatic | HR+, HER2- | 2nd-line | Capivasertib | Fulvestrant | Placebo+ Fulvestrant | Non-steroidal AI therapy failed, Grade of Recommendation I: Steroidal AI+ Chidamide OR Fulvestrant+ Abemaciclib OR Fulvestrant + Palbociclib; Grade of Recommendation II: Steroidal AI+ CDK4/6 inhibitor; Fulvestrant; Steroidal AI+ Everolimus Steroidal AI therapy failed, Grade of Recommendation I: Fulvestrant+ Abemaciclib OR Fulvestrant + Palbociclib; Grade of Recommendation II: Fulvestrant OR Non-steroidal AI+ CDK4/6 inhibitor |
| 48 | CTR20211931 | Jun-21 | III | China-only | Breast | Locally advanced or metastatic | HR+, HER2- | 2nd-line | GB491 (Lerociclib) | Fulvestrant | Placebo+ Fulvestrant | Non-steroidal AI therapy failed, Grade of Recommendation I: Steroidal AI+ Chidamide OR Fulvestrant+ Abemaciclib OR Fulvestrant + Palbociclib; Grade of Recommendation II: Steroidal AI+ CDK4/6 inhibitor; Fulvestrant; Steroidal AI+ Everolimus Steroidal AI therapy failed, Grade of Recommendation I: Fulvestrant+ Abemaciclib OR Fulvestrant + Palbociclib; Grade of Recommendation II: Fulvestrant OR Non-steroidal AI+ CDK4/6 inhibitor |
| 49 | CTR20212307 | Aug-21 | III | China-only | Breast | Locally advanced or metastatic | HR+, HER2- | 2nd-line | Birociclib | Fulvestrant | Placebo+ Fulvestrant | Non-steroidal AI therapy failed, Grade of Recommendation I: Steroidal AI+ Chidamide OR Fulvestrant+ Abemaciclib OR Fulvestrant + Palbociclib; Grade of Recommendation II: Steroidal AI+ CDK4/6 inhibitor; Fulvestrant; Steroidal AI+ Everolimus Steroidal AI therapy failed, Grade of Recommendation I: Fulvestrant+ Abemaciclib OR Fulvestrant + Palbociclib; Grade of Recommendation II: Fulvestrant OR Non-steroidal AI+ CDK4/6 inhibitor |
| 50 | CTR20212304 | Aug-21 | III | China-only | Breast | Postmenopausal advanced or metastatic | ER+, HER2+ | 2nd-line | Hemay022 | AI (Letrozole/Exemestane) | Lapatinib+ Capecitabine | Grade of Recommendation I: Pyrotinib+ Capecitabine; Grade of Recommendation II: Trastuzumab Emtansine OR Lapatinib+ Capecitabine |
| 51 | CTR20213271 | Nov-21 | III | China-only | Breast | Locally advanced or metastatic | HR+, HER2- | 2nd-line | TQB3616 | Fulvestrant | Placebo+ Fulvestrant | Non-steroidal AI therapy failed, Grade of Recommendation I: Steroidal AI+ Chidamide OR Fulvestrant+ Abemaciclib OR Fulvestrant + Palbociclib; Grade of Recommendation II: Steroidal AI+ CDK4/6 inhibitor; Fulvestrant; Steroidal AI+ Everolimus Steroidal AI therapy failed, Grade of Recommendation I: Fulvestrant+ Abemaciclib OR Fulvestrant + Palbociclib; Grade of Recommendation II: Fulvestrant OR Non-steroidal AI+ CDK4/6 inhibitor |
| 52 | CTR20213307 | Dec-21 | III | China-only | Breast | Advanced | HR+, HER2- | 2nd-line | FCN-437c | Fulvestrant± Goserelin | Placebo+ Fulvestrant± Goserelin | Non-steroidal AI therapy failed, Grade of Recommendation I: Steroidal AI+ Chidamide OR Fulvestrant+ Abemaciclib OR Fulvestrant + Palbociclib; Grade of Recommendation II: Steroidal AI+ CDK4/6 inhibitor; Fulvestrant; Steroidal AI+ Everolimus Steroidal AI therapy failed, Grade of Recommendation I: Fulvestrant+ Abemaciclib OR Fulvestrant + Palbociclib; Grade of Recommendation II: Fulvestrant OR Non-steroidal AI+ CDK4/6 inhibitor |
| 53 | CTR20182046 | Jun-18 | III | International multicenter | Breast | Stage II-III | HER2-&ER-&PgR- | adjuvant | Atezolizumab | TAC (Paclitaxel→Doxorubicin/Epirubicin+ Cyclophosphamide) | TAC (Paclitaxel→Doxorubicin/Epirubicin+ Cyclophosphamide) | Grade of Recommendation I: AC-T; Grade of Recommendation II: TAC 10 OR FEC-T |
| 54 | CTR20201563 | Jul-20 | III | China-only | Breast | Stage II-III | ER-, PR-, HER2- | neoadjuvant | Camrelizumab | Carboplatin/(Epirubicin+ Cyclophosphamide)+ Paclitaxel (Albumin Bound) | Placebo+ Carboplatin/ (Epirubicin+ Cyclophosphamide)+ Paclitaxel (Albumin Bound) | Grade of Recommendation I: TAC OR AT; Grade of Recommendation II: AC-T |
| 55 | CTR20211227 | Sep-21 | III | China-only | Cervix | Recurrent or metastatic | / | 1st-line | Camrelizumab | Famitinib | Platinum-based Standard Chemotherapy (Paclitaxel+ Carboplatin/Cisplatin) | Principle strategy: Cisplatin/Carboplatin+ Paclitaxel+ Bevacizumab; Optional strategy: Cisplatin/Carboplatin+ Paclitaxel; Topotecan+ Paclitaxel+ Bevacizumab |
| 56 | CTR20202685 | Aug-21 | III | International multicenter | Head and Neck | Recurrent or metastatic | / | 2nd-line | AN2025 (Buparlisib) | Paclitaxel | Paclitaxel | Grade of Recommendation I: Nivolumab; Grade of Recommendation II: Pembrolizumab; Methotrexate; Docetaxel; Paclitaxel; Cetuximab |
| 57 | CTR20212957 | Apr-21 | III | International multicenter | Liver | Locally advanced or metastatic, and/or unresectable | / | 2nd-line | Atezolizumab | Lenvatinib/Sorafenib | Lenvatinib/Sorafenib | Grade of Recommendation I: Regorafenib OR PD-1 McAb(Nivolumab/Pembrolizumab/Camrelizumab) OR Apatinib (1A); Grade of Recommendation II: Ramucirumab (AFP≥ 400ng/ml ) OR Cabozantinib OR Camrelizumab+ FOLFOX4/Apatinib OR Sorafenib; chemotherapy |
| 58 | CTR20211262 | May-21 | III | China-only | Lung-NSCLC | Locally advanced or metastatic | EGFR+ | 1st-line | Almonertinib | / | Platinum-Doublet Chemotherapy (Pemetrexed+ Cisplatin/Carboplatin) | Grade of Recommendation I: Gefitinib OR Erlotinib OR Icotinib OR Afatinib OR Dacomitinib OR Osimertinib; Grade of Recommendation II: Gefitinib/Erlotinib+ chemotherapy OR Erlotinib+Bevacizumab OR Platinum-Doublet Chemotherapy± Bevacizumab |
| 59 | CTR20201863 | Sep-20 | III | International multicenter | Oesophagus | Locally advanced, recurrent or metastatic | / | 1st-line | Tiragolumab | Atezolizumab+ Paclitaxel+ Cisplatin | Paclitaxel+ Cisplatin | Grade of Recommendation I: Fluorouracil (5-FU OR Capecitabine OR S-1)+ Cisplatin; Grade of Recommendation II: Fluorouracil+ Irinotecan OR Paclitaxel/Docetaxel/Vinorelbine+ Cisplatin/Nedaplatin |
| 60 | CTR20202224 | Oct-20 | III | China-only | Urothelium | Intermediate-risk | / | adjuvant | APL-1202 | / | Intravesical Epirubicin | Grade of Recommendation I: Single immediate instillation chemotherapy+ Intravesical Bacillus Calmette-Guerin 1 year (Priority); Grade of Recommendation I: Immediate instillation of chemotherapy+ Chemotherapy OR Intravesical Bacillus Calmette-Guerin |

***Abbreviations:***

*5-FU, 5-fluorouracil; A, anthracyclines; ADT, androgen deprivation therapy; AI, aromatase inhibitor; ALK, anaplastic lymphoma kinase; C, cyclophosphamide; CCRT, concurrent chemoradiotherapy; CDK4/6, cyclin-dependent kinase4/6; ctDNA, circulating tumor deoxyribonucleic acid; DLBCL, diffuse large B-cell lymphoma; EGFR, epidermal growth factor receptor; ER, estrogen receptor; FEC, 5-FU, epirubicin, and cyclophosphamide; FIGO, federation international of gynecology and obstetrics; FOLFIRI, leucovorin, fluorouracil, and irinotecan hydrochloride; FOLFOX, folinic acid, fluorouracil, and oxaliplatin; GemOx, gemcitabine and oxaliplatin; HER2,human epidermal growth factor receptor 2; HR, hormone receptor; IRB, institutional review board; mFOLFOX6, oxaliplatin, leucovorin, and 5-fluorouracil; NSCLC, non-small cell lung cancer; PARP, poly ADP-ribose polymerase; PD-1, programmed cell death 1;PD-L1, programmed cell death-ligand 1; PR, progesterone receptor; R, Rituximab; R2, rituximab and lenalidomide; R-CHOP, R-CHOP, rituximab, cyclophosphamide, doxorubicin, vincristine, and prednisone; RCVP, rituximab, cyclophosphamide, vincristine, and prednisone; R-DA-EPOCH, rituximab, etoposide, prednisone, vincristine, cyclophosphamide, and doxorubicin; R-DHAP, rituximab, dexamethasone, cisplatin, and cytarabine; R-ESHAP, rituximab, etoposide, methylprednisolone, and cisplatin; R-GDP, rituximab, gemcitabine, cisplatin, and dexamethasone; R-ICE, rituximab, ifosfamide, carboplatin, and etoposide; R-MINE, rituximab, ifosfamide, mitoxantrone, and etoposide; S-1, tegafur, gimeracil, and oteracil porassium; SCLC, small cell lung cancer; SOX, S-1 and oxaliplatin; T, taxoids; TACE, transarterial chemoembolization; TKI, tyrosine kinase inhibitor; XELOX, capecitabine and oxaliplatin; XP, capecitabine and cisplatin,*

***Note:***

*Category 1. The control was not recommended by the concerned clinical practice guideline for a specific indication issued prior to the trial’s ethical approval*

*Category 2. The control drug or regimen was specified in the guideline but not the recommended regimen*

*Category 3. The control arm was among the recommended list of therapies but there existed a drug (or drugs) with a higher level of recommendation*

## Table D. Primary endpoints used in sample randomized oncology trials with published results

| **Primary Endpoint** | **Trials, Number (%)** |
| --- | --- |
| **Total** | **105 (100.0)** |
| PFS | 56 (53.3) |
| OS | 24 (22.9) |
| PFS and OS (co-primary endpoints) | 14 (13.3) |
| DFS | 3 (2.9) |
| EFS | 2 (1.9) |
| sustained testosterone suppression to castrate levels (<50 ng per deciliter) through 48 weeks | 1 (1) |
| IDFS | 1 (1) |
| tpCR | 1 (1) |
| Very good partial response or better rates | 1 (1) |
| ORR | 1 (1) |
| PFS and pCR (co-primary endpoints) | 1 (1) |

***Abbreviations:***

*DFS, disease-free survival; EFS, event-free survival; IDFS, invasive disease-free survival; ORR, objective response rate; OS, overall survival; pCR, pathological complete response; PFS, progression-free survival; tpCR, total pathological complete response.*

## Table E. Primary endpoint results and statistical significance of sample randomized oncology trials

| **No.** | **CTR** | **NCT** | **Primary Endpoint(s)** | **Results** | **HR (95% CI)** | **P-value** | **Statistically significant results** | **Reference PMID** |
| --- | --- | --- | --- | --- | --- | --- | --- | --- |
| **Optimal & Potentially Optimal Control** | | |  |  |  |  |  |  |
| 1 | CTR20160233 | NCT02489318 | PFS OS | 24m PFS% 68.2% vs 47.5% 24m OS% 82.4% vs 73.5% | 0.48 [0.39, 0.60] 0.67 [0.51, 0.89] | p<0.001 0.005 | Y | 31150574 |
| 2 | CTR20160293 | NCT02178956 | OS | 6.93 vs. 7.36 | 1.01 [0.86, 1.20] | 0.8596 | N | 35833783 |
| 3 | CTR20160361 | NCT02799602 | OS | NR vs 48.9 | 0.68 [0.57, 0.80] | p<0.001 | Y | 35179323 |
| 4 | CTR20160365 | NCT02763566 | PFS | NR vs 14.7 | 0.499 [0.346, 0.719] | 0.0001 | Y | 33149768 |
| 5 | CTR20160366 | NCT02896855 | PFS | 14.5 vs 12.4 | 0.69 [0.49, 0.99] | Not reported | Y | 32564260 |
| 6 | CTR20160367 | NCT02838420 | PFS | NR vs 11.1 | 0.22 [0.13, 0.38] | p<0.0001 | Y | 30981696 |
| 7 | CTR20160574 | NCT02898077 | PFS OS | PFS: 4.14 vs 3.15 OS: 8.71 vs 7.92 | 0.765 [0.613, 0.955] 0.963 [0.771, 1.203] | 0.0184 0.7426 | Y | 34626550 |
| 8 | CTR20160587 | NCT03019588 | PFS OS | PFS: 2 vs 4 OS: 8 vs 8 | 1.62 [1.04, 2.52] 0.99 [0.63, 1.54] | Not reported Not reported | N | 34878659 |
| 9 | CTR20160894 | NCT02340221 | PFS | 7.4 vs 5.4 | 0.70 [0.56, 0.89] | 0.0037 | Y | 33186740 |
| 10 | CTR20160994 | NCT02409342 | OS | 20.2 vs 13.1 | 0.59 [0.40, 0.89] | 0.01 | Y | 32997907 |
| 11 | CTR20170044 | NCT02775435 | PFS OS | PFS: 6.4 vs 4.8 OS: 15.9 vs 11.3 | 0.56 [0.45, 0.70] 0.64 [0.49, 0.85] | p<0.001 p<0.001 | Y | 30280635 |
| 12 | CTR20170322 | NCT03134872 | PFS | 11.3 vs 8.3 | 0.60 [0.45, 0.79] | one-sided p=0.0001 | Y | 33347829 |
| 13 | CTR20170340 | NCT02477826 | PFS OS | PFS: 7.2 vs 5.5 OS results not reported | 0.58 [0.41, 0.81] | p<0.001 | Y | 29658845 |
| 14 | CTR20170371 | NCT02872116 | PFS OS | PFS: 7.7 vs 6.9 OS: 13.8 vs 11.6 | 0.77 [0.68, 0.87] 0.80 [0.68, 0.94] | Not reported 0.0002 | Y | 34102137 |
| 15 | CTR20170380 | NCT03150875 | OS | 11.79 vs 8.25 | 0.74 [0.56, 0.96] | 0.025 | Y | 36336841 |
| 16 | CTR20170486 | NCT02753127 | OS | 14.3 vs 13.8 | 0.976 [0.854, 1.117] | Nominal one-sided p=0.74 | N | 36503738 |
| 17 | CTR20170599 | NCT03024996 | DFS | 57.2 vs 49.5 | 0.93 [0.75, 1.15] | 0.5 | N | 36099926 |
| 18 | CTR20170661 | NCT02668653 | OS | 31.9 vs 15.1 | 0.78 [0.62, 0.98] | 0.032 | Y | 37116523 |
| 19 | CTR20170694 | NCT02538666 | OS | 9.2 vs 9.6 | 0.92 [0.75, 1.12] | 0.37 | N | 33683919 |
| 20 | CTR20170772 | NCT03234972 | PFS | NR vs 6.3 | 0.28 [0.17, 0.47] | p<0.00001 | Y | 34108127 |
| 21 | CTR20170919 | NCT02767804 | PFS | 25.8 vs 12.7 | 0.51 [0.35, 0.72] | p<0.001 | Y | 34473194 |
| 22 | CTR20170961 | NCT02577406 | OS | 6.5 vs 6.2 | 0.86 [0.67, 1.10] | 0.23 | N | 35714312 |
| 23 | CTR20171002 | NCT03217812 | very good partial response or better rates | 74.0% vs 43.2% | 3.57 [1.99, 6.43] | p<0.0001 | Y | 37024420 |
| 24 | CTR20171026 | NCT03430843 | OS | 8.6 vs 6.3 | 0.70 [0.57, 0.85] | one-sided p=0.0001 | Y | 35442766 |
| 25 | CTR20171112 | NCT03358875 | OS | 17.2 vs 11.9 (interim analysis) 16.9 vs 11.9 (final analysis) | 0.64 [0.53, 0.78] 0.66 [0.56, 0.79] | p<0.0001 | Y | 36184068 |
| 26 | CTR20171115 | NCT03125902 | PFS | 6.0 vs 5.7 | 0.82 [0.60, 1.12] | 0.2 | N | 34219000 |
| 27 | CTR20171172 | NCT03155997 | IDFS | 2yr IDFS% 92.2% vs 88.7% | 0.75 [0.60, 0.93] | 0.01 | Y | 32954927 |
| 28 | CTR20171227 | NCT03143153 | PFS OS | OS: 6.9 vs 4.4 PFS: 15.4 vs 9.1 | 0.65 [0.46, 0.92] 0.54 [0.37, 0.80] | 0.002 p<0.001 | Y | 35108470 |
| 29 | CTR20171499 | NCT03069352 | OS | 7.2 vs 4.1 8.4 vs 4.1 | 0.75 [0.52, 1.07] 0.70 [0.50, 0.99] | 0.11 0.04 | Y | 32219442 |
| 30 | CTR20171524 | NCT03072238 | PFS | 18.5 vs 16.5 | 0.77 [0.61, 0.98] | 0.034 | Y | 34246347 |
| 31 | CTR20171624 | NCT03052608 | PFS | 12m PFS% 78% vs 39% | 0.28 [0.19, 0.41] | p<0.001 | Y | 33207094 |
| 32 | CTR20171629 | NCT03191786 | OS | 10.3 vs 9.2 | 0.78 [0.63, 0.97] | 0.028 | Y | 37423228 |
| 33 | CTR20171642 | NCT03189719 | PFS OS | PFS: 6.3 vs 5.8 OS: 13.9 vs 8.8 | 0.65 [0.54, 0.78] 0.57 [0.43, 0.75] | p<0.0001 p<0.0001 | Y | 34454674 |
| 34 | CTR20180032 | NCT03663205 | PFS | 9.7 vs 7.6 | 0.645 [0.462, 0.902] | 0.0044 | Y | 34033975 |
| 35 | CTR20180072 | NCT03138512 | DFS | NR vs 50.7 | 0.92 [0.71, 1.19] | 0.53 | N | 36774933 |
| 36 | CTR20180090 | NCT03043872 | OS | 13.0 vs 10.3 | 0.73 [0.59, 0.91] | 0.0047 | Y | 31590988 |
| 37 | CTR20180162 | NCT02987543 | PFS | 7.4 vs 3.6 | 0.34 [0.25, 0.47] | p<0.001 | Y | 32343890 |
| 38 | CTR20180292 | NCT03594747 | PFS | 7.6 vs 5.5 | 0.524 [0.370, 0.742] | p<0.001 | Y | 33792623 |
| 39 | CTR20180560 | NCT03520478 | PFS OS | PFS: NR vs 25.1 OS: NR vs NR | 0.44 [0.33, 0.58] 0.58 [0.44, 0.77] | p<0.0001 0.0001 | Y | 36075260 |
| 40 | CTR20180636 | NCT03164616 | PFS OS | PFS: 5.5 vs 4.8 OS: 13.3 vs 11.7 | 0.74 [0.62, 0.89] 0.86 [0.72, 1.02] | 0.0009 0.0758 | Y | 36327426 |
| 41 | CTR20180888 | NCT03516084 | PFS OS | PFS: 1.54 vs 1.36 OS: 9.92 vs 11.43 | 0.66 [0.46, 0.95] 1.03 [0.62, 1.73] | 0.0242 0.9052 | Y | 33915252 |
| 42 | CTR20180914 | NCT03215706 | OS | 14.1 vs 10.7 (interim analysis) 15.6 vs 10.9 (updated OS) | 0.69 [0.55, 0.87] 0.66 [0.55, 0.90] | 0.00065 Not reported | Y | 33476593 |
| 43 | CTR20180941 | NCT03588091 | tpCR | 41.0% vs 22.0% | Not reported | one-sided p<0.0001 | Y | 36575513 |
| 44 | CTR20180975 | NCT03607539 | PFS | 8.9 vs 5.0 | 0.482 [0.362, 0.643] | p<0.00001 | Y | 32781263 |
| 45 | CTR20181013 | NCT03783442 | OS | 17.2 vs 10.6 | 0.66 [0.54, 0.80] | one-sided p<0.0001 | Y | 37080222 |
| 46 | CTR20181228 | NCT03061812 | OS | 6.3 vs 8.6 | 1.46 [1.17, 1.82] | Not reported | N | 33607312 |
| 47 | CTR20181229 | NCT03033511 | PFS OS | PFS: 4.0 vs 1.4 OS: 8.5 vs 9.8 | 0.48 [0.39, 0.59] 1.07 [0.84, 1.36] | p<0.001 Not reported | Y | 33823285 |
| 48 | CTR20181308 | NCT03748134 | OS | 16.7 vs 12.5 | 0.63 [0.51, 0.78] | p<0.001 | Y | 35440464 |
| 49 | CTR20181396 | NCT03274492 | PFS | 76.7% vs 70.2% | 0.73 [0.57, 0.95] | 0.02 | Y | 34904799 |
| 50 | CTR20181413 | NCT03085095 | sustained testosterone suppression to castrate levels (<50 ng per deciliter) through 48 weeks | 96.7% vs 88.8% | Not reported | p<0.001 | Y | 32469183 |
| 51 | CTR20181429 | NCT03728556 | PFS | 9.0 vs 5.8 | 0.64 [0.48, 0.85] | 0.0026 | Y | 35038429 |
| 52 | CTR20181437 | NCT03629925 | PFS | 5.5 vs 4.9 | 0.536 [0.422, 0.681] | p<0.00001 | Y | 34048947 |
| 53 | CTR20181452 | NCT03789604 | PFS | 7.8 vs 4.9 (interim analysis) 9.0 vs 4.9 (final analysis) | 0.50 [0.39, 0.64] 0.48 [0.39, 0.60] | p<0.0001 | Y | 35038432 |
| 54 | CTR20181611 | NCT03668496 | PFS | 8.5 vs 4.9 | 0.37 [0.29, 0.47] | p<0.0001 | Y | 34923163 |
| 55 | CTR20181718 | NCT03691090 | PFS OS | PFS: 6.9 vs 5.6 OS: 15.3 vs 12.0 | 0.56 [0.46, 0.68] 0.70 [0.56, 0.88] | 1-sided p<0.001 1-sided p=0.001 | Y | 34519801 |
| 56 | CTR20181746 | NCT03594747 | PFS | 7.6 vs 5.5 | 0.524 [0.370, 0.742] | p<0.001 | Y | 33792623 |
| 57 | CTR20181951 | NCT03849768 | PFS | 19.3 vs 9.9 | 0.46 [0.36, 0.60] | p<0.0001 | Y | 35580297 |
| 58 | CTR20182176 | NCT03711305 | OS | 15.2 vs 12.8 | 0.72 [0.58, 0.90] | one-sided p=0.0017 | Y | 35576956 |
| 59 | CTR20182270 | NCT03337724 | PFS | 9.3 vs 9.3 | 1.00 [0.71, 1.40] | 0.997 | N | 34860318 |
| 60 | CTR20182519 | NCT03787992 | PFS | 20.8 vs 11.1 | 0.44 [0.34, 0.58] | p<0.0001 | Y | 35662408 |
| 61 | CTR20182528 | NCT03764293 | PFS | 5.6 vs 3.7 | 0.52 [0.41, 0.65] | one-sided p<0.0001 | Y | 37499670 |
| 62 | CTR20182530 | NCT03794440 | PFS OS | PFS: 4.6 vs 2.8 OS: NR vs 10.4 | 0.56 [0.46, 0.70] 0.57 [0.43, 0.75] | p<0.0001 p<0.0001 | Y | 34143971 |
| 63 | CTR20182550 | NCT03802240 | PFS | 6.9 vs 4.3 | 0.46 [0.34, 0.64] | p<0.0001 | Y | 35908558 |
| 64 | CTR20182559 | NCT03802240 | PFS | 6.9 vs 4.3 | 0.46 [0.34, 0.64] | p<0.0001 | Y | 35908558 |
| 65 | CTR20190098 | NCT03734016 | ORR | 78.3% vs 62.5% | Not reported | p<0.001 | Y | 36395435 |
| 66 | CTR20190147 | NCT03856411 | PFS | 8.3 vs 5.6 | 0.58 [0.44, 0.77] 0.49 [0.39, 0.61] | 0.0001 p<0.0001 | Y | 36206498 |
| 67 | CTR20190159 | NCT03732820 | PFS | 24.8 vs 16.6 | 0.66 [0.54, 0.81] | p<0.001 | Y | NA |
| 68 | CTR20190258 | NCT03504397 | PFS | 12.94 vs 12.65 | 0.75 [0.60, 0.94] | 0.0066 | Y | 37068504 |
| 69 | CTR20190261 | NCT03653507 | PFS | 8.21 vs 6.80 | 0.687 [0.544, 0.866] | 0.0007 | Y | 37524953 |
| 70 | CTR20190416 | NCT03336333 | PFS | NR vs NR | 0.42 [0.28, 0.63] | p<0.0001 | Y | 35810754 |
| 71 | CTR20190619 | NCT03927456 | PFS | 15.7 vs 7.2 | 0.42 [0.31, 0.58] | one-sided p<0.0001 | Y | 34737452 |
| 72 | CTR20190754 | NCT04063163 | OS | 15.4 vs 10.9 | 0.63 [0.49, 0.82] | p<0.001 | Y | 36166026 |
| 73 | CTR20190911 | NCT03958890 | PFS | 5.8 vs 5.3 | 0.60 [0.48, 0.75] | p<0.0001 | Y | 36732627 |
| 74 | CTR20190962 | NCT03966898 | PFS | 30.6 vs 18.2 | 0.51 [0.38, 0.69] | one-sided log-rank p<0.0001 | Y | 37182538 |
| 75 | CTR20191289 | NCT04009317 | PFS | 24.87 vs 11.60 | 0.47 [0.34, 0.64] | p<0.0001 | Y | 37574511 |
| 76 | CTR20191443 | NCT03395197 | PFS | NR vs 21.9 | 0.63 [0.51, 0.78] | p<0.0001 | Y | 37285865 |
| 77 | CTR20191452 | NCT03409614 | OS | 21.9 vs 13.0 | 0.71 [0.53, 0.93] | 0.014 | Y | 36008722 |
| 78 | CTR20191453 | NCT03088540 | PFS | 8.2 vs 5.7 | 0.54 [0.43, 0.68] | p<0.0001 | Y | 33581821 |
| 79 | CTR20191551 | NCT03764293 | PFS | 5.6 vs 3.7 | 0.52 [0.41, 0.65] | one-sided p<0.0001 | Y | 37499670 |
| 80 | CTR20191730 | NCT03734029 | PFS | 10.1 vs 5.4 | 0.51 [0.40, 0.64] | p<0.001 | Y | 35665782 |
| 81 | CTR20191819 | NCT03755791 | PFS | 6.8 vs 4.2 | 0.63 [0.44, 0.91] | 0.0012 | Y | 35798016 |
| 82 | CTR20192035 | NCT03268954 | EFS | 17.7 vs 15.7 | 0.968 [0.757, 1.238] | 0.557 | N | 35728048 |
| 83 | CTR20192356 | NCT04206072 | PFS | 22.1 vs 13.8 | 0.49 [0.36, 0.68] | p<0.0001 | Y | 37244266 |
| 84 | CTR20192713 | NCT04003636 | OS | 12.7 vs 10.9 | 0.83 [0.72, 0.95] | one-sided p=0.0034 | Y | 37075781 |
| 85 | CTR20211721 | NCT03555422 | PFS | 5.7 vs 3.8 | 0.76 [0.54, 1.08] | 0.126 | N | 37669480 |
| 86 | CTR20212889 | NCT03581786 | PFS | 11.7 vs 8.0 | 0.52 [0.36, 0.74] | 0.0003 | Y | 34341578 |
| 87 | CTR20170690 | NCT03016312 | OS | 15.2 vs 16.6 | 1.12 [0.91, 1.37] | 0.28 | N | 35013615 |
| 88 | CTR20171498 | NCT02993523 | OS | 14.7 vs 9.6 | 0.66 [0.52, 0.85] | p<0.001 | Y | 32786187 |
| 89 | CTR20181749 | NCT03416179 | OS | 12.7 vs 12.2 | 1.05 [0.782, 1.408] | two-sided p=0.749 | N | 37604981 |
| 90 | CTR20181920 | NCT03173248 | EFS | 12m EFS% 37% vs 12% | 0.33 [0.16, 0.69] | 0.002 | Y | 35443108 |
| 91 | CTR20182342 | NCT03596866 | PFS | 19.3 vs 19.2 | 0.97 [0.66, 1.42] | 0.8672 | N | 37574132 |
| 92 | CTR20190378 | NCT03529110 | PFS | 12m PFS% 75.8% vs 34.1% | 0.28 [0.22, 0.37] | p<0.001 | Y | 35320644 |
| 93 | CTR20192195 | NCT03748641 | PFS | 16.5 vs 13.7 | 0.73 [0.56, 0.96] | 0.022 | Y | 36952634 |
| **Suboptimal Control** | | |  |  |  |  |  |  |
| 94 | CTR20160121 | NCT02966093 | PFS | 23.0 vs 3.7 | 0.16 [0.10, 0.26] | p<0.0001 | Y | 34326132 |
| 95 | CTR20170038 | NCT02312258 | PFS | 17.4 v 9.4 | 0.659 [0.542, 0.801] | p<0.001 | Y | 33021870 |
| 96 | CTR20171558 | NCT03538171 | PFS | 6.32 vs 3.72 | 0.76 [0.58, 0.98] | 0.046 | Y | 37250148 |
| 97 | CTR20180183 | NCT02743494 | DFS | 22.4 vs 11.0 | 0.69 [0.56, 0.86] | p<0.001 | Y | 33789008 |
| 98 | CTR20180191 | NCT03602495 | PFS | 12.9 vs 6.4 | 0.39 [0.25, 0.61] | p<0.0001 | Y | 37184934 |
| 99 | CTR20180718 | NCT02312258 | PFS | 17.4 vs 9.4 | 0.659 [0.542, 0.801] | p<0.001 | Y | 33021870 |
| 100 | CTR20180929 | NCT02998528 | PFS pCR | PFS: 31.6 vs 20.8 pCR: 24.0% vs 2.2% | HR 0.63 [0.43, 0.91] OR 13.94 [3.49, 55.75] | 0.005 p<0.001 | Y | 35403841 |
| 101 | CTR20190294 | NCT03863860 | PFS | 12.9 vs 5.5 | 0.25 [0.17, 0.36] | one-sided p<0.0001 | Y | 35404684 |
| 102 | CTR20190968 | NCT03802240 | PFS | 6.9 vs 4.3 | 0.46 [0.34, 0.64] | p<0.0001 | Y | 35908558 |
| 103 | CTR20190972 | NCT03802240 | PFS | 6.9 vs 4.3 | 0.46 [0.34, 0.64] | p<0.0001 | Y | 35908558 |
| 104 | CTR20200467 | NCT03538171 | PFS | 6.32 vs 3.72 | 0.76 [0.58, 0.98] | 0.046 | Y | 37250148 |
| 105 | CTR20211545 | NCT04305496 | PFS | 7.2 vs 3.6 | 0.60 [0.51, 0.71] | p<0.001 | Y | 37256976 |

***Abbreviations:***

*CI, confidence interval; DFS, disease-free survival; EFS, event-free survival; IDFS, invasive disease-free survival; N, no; NR, not reached; ORR, objective response rate; OS, overall survival; pCR, pathological complete response; PFS, progression-free survival; tpCR, total pathological complete response; Y, yes.*

## Fig A. Use of suboptimal control arm in randomized controlled oncology trials in China, by applicable guideline type

***Abbreviations:*** *CSCO, Chinese Society of Clinical Oncology.*

***Note:*** *No statistically significant difference was observed in the proportion of suboptimal control use among the two types of guidelines (13.8% vs 11.3%, Fisher’s exact test P=0.614).*

## Table F. Guideline published duration before the ethical approval of sample randomized controlled trials

| **Guideline published duration before trial ethic approval (month)** | **All (%), N=453** | **Trials, Number (%)** | |
| --- | --- | --- | --- |
|  |  | **Optimal & potentially optimal control arm, N=393** | **Suboptimal control arm, N=60** |
| <1 | 30 (6.6) | 27 (6.9) | 3 (5.0) |
| 1-2 | 34 (7.5) | 29 (7.4) | 5 (8.3) |
| 2-3 | 34 (7.5) | 28 (7.1) | 6 (10.0) |
| 3-4 | 25 (5.5) | 21 (5.3) | 4 (6.7) |
| 4-5 | 30 (6.6) | 23 (5.9) | 7 (11.7) |
| 5-6 | 27 (6.0) | 22 (5.6) | 5 (8.3) |
| 6-7 | 24 (5.3) | 20 (5.1) | 4 (6.7) |
| 7-8 | 19 (4.2) | 16 (4.1) | 3 (5.0) |
| 8-9 | 34 (7.5) | 26 (6.6) | 8 (13.5) |
| 9-10 | 23 (5.1) | 20 (5.1) | 3 (5.0) |
| 10-11 | 20 (4.4) | 19 (4.8) | 1 (1.7) |
| 11-12 | 28 (6.2) | 26 (6.6) | 2 (3.3) |
| >12 | 125 (27.6) | 116 (29.5) | 9 (15.0) |
