# Supplementary material for: Use of suboptimal control arms in randomized clinical trials of investigational cancer drugs in China, 2016–2021: An observational study
Source: PLoS Med. 2023 Dec 12;20(12):e1004319. doi: 10.1371/journal.pmed.1004319 (PMC10715645; doi:10.1371/journal.pmed.1004319)
Supplement: Attachment — Submitted filename: 1_Author Response_clean.docx [file pmed.1004319.s003.docx]

**REQUEST FROM THE EDITORS:**

**GENERAL**

Please respond to all editor and reviewer comments detailed below in full.

We recommend that you refer to our checklist of formatting requirements before resubmitting in order to expedite your manuscript through the technical checks. Download the formatting checklist (PDF).

**Authors:** Thank you. This revision referred to the formatting checklist as suggested.

**TITLE**

Please revise your title according to PLOS Medicine's style. Your title must be nondeclarative and not a question. It should begin with main concept if possible. "Effect of" should be used only if causality can be inferred, i.e., for an RCT. Please place the study design ("A randomized controlled trial," "A retrospective study," "A modelling study," etc.) in the subtitle (ie, after a colon).

**Authors:** We added the study design to the title as suggested. The revised manuscript is titled “Use of Suboptimal Control Arms in Randomized Clinical Trials of Investigational Cancer Drugs in China, 2016-2021: An Observational Study”.

**COMPETING INTERESTS**

All authors must declare their relevant competing interests per the PLOS policy, which can be seen here:

https://journals.plos.org/plosmedicine/s/competing-interests

For authors with ties to industry, please indicate whether any of the interests has a financial stake in the results of the current study.

**Authors:** Thanks. Following the PLOS policy, we added the *Competing Interests* in the Title Page. In Page 2 Line 27, we wrote:

*“I have read the journal’s policy and the authors of this manuscript have the following competing interests: X.G. acknowledged receiving grants from the Research on Equitable Access and Rational Use of Medicines sponsored by the Peking University Health Science Center. X.G. and L.S. reported receiving grants from the National Natural Science Foundation of China outside the submitted work. The funders had no role in study design, data collection and analysis, decision to publish, or preparation of the manuscript. Other authors have declared that no competing interests exist.”*

**ABSTRACT**

**Abstract methods and findings:**

If not reporting p values for the purpose of transparent data reporting, please clearly state the reasons why not. Please report p values as <0.001 and where higher the exact as p value, p=0.002, for example.

**Authors:** We reported exact p values in the Abstract methods and findings. In Page 4 Line 58, we wrote:

*“For the 105 trials with publicly available results, no statistically significant difference was observed between the use of a suboptimal control and concluding positive on the primary endpoint (100.0% vs. 83.9%, p=0.208).”*

Please include any important dependent variables that are adjusted for in the analyses.

**Authors:** We reported all adjusted variables in the regression model as suggested. In Page 3 Line 54, we wrote:

*“After adjusting for the year of ethical approval, region of participant recruitment, line of therapy, and cancer site, second-line therapies (adjusted odds ratio [aOR]=2.7, 95%CI [1.2, 5.9]), adjuvant therapies (aOR= 8.9, 95%CI [3.4, 23.1]), maintenance therapies (aOR=5.2, 95%CI [1.6-17.0]), and trials recruiting participants in China only (aOR=4.1, 95%CI [2.1, 8.0]) were more likely to adopt a suboptimal control.”*

Line 28 – please remove the word retrospective. Please check and amend throughout all subsections of the manuscript to refer to your study as, ‘observational’.

**Authors:** We checked and removed ‘retrospective’ throughout the manuscript. Study categorization was restricted to ‘observational’. In Page 3 Line 43, we wrote:

*“This observational study included randomized controlled trials of cancer drugs, authorized by concerned Chinese institutional review boards between 2016 and 2021, supporting investigational new drug applications of these drugs in China.”*

In the last sentence of the Abstract Methods and Findings section, please describe the main limitation(s) of the study's methodology.

**Authors:** We added the main limitation to the last sentence of the Abstract Methods and Findings section. In Page 4 Line 60, we wrote:

*“The main limitation of this study is its reliance on clinical guidelines that could vary across cancer types and time in assessing the quality of the control groups.”*

**Abstract Conclusions:**

Suggest beginning, ‘In this study…’

**Authors:** Thanks. We modified our expression in *Conclusion* in the abstract as suggested. In Page 4 Line 64, we wrote:

*“In this study, over one-eighth of randomized trials of cancer drugs registered to apply for regulatory approval in China used a suboptimal comparator.”*

The final statement could be more powerful, without over stating your results. Your data could have implications for clinical trial design and regulation of pharmaceutical companies. I think that’s what you mean by ‘collaborative efforts’ but it does not explicitly make the point and that is the real message.

**Authors:** Sorry for any confusion. We modified the final statement to make it more explicit. In Page 4 Line 64, we wrote:

*“Our results highlight the necessity to refine the design of randomized trials to generate optimal clinical evidence for new cancer therapies.”*

**AUTHOR SUMMARY**

At this stage, we ask that you include a short, non-technical Author Summary of your research to make findings accessible to a wide audience that includes both scientists and non-scientists. The authors summary should consist of 2-3 succinct bullet points under each of the following headings:

• Why Was This Study Done? Authors should reflect on what was known about the topic before the research was published and why the research was needed.

• What Did the Researchers Do and Find? Authors should briefly describe the study design that was used and the study’s major findings. Do include the headline numbers from the study, such as the sample size and key findings.

• What Do These Findings Mean? Authors should reflect on the new knowledge generated by the research and the implications for practice, research, policy, or public health. Authors should also consider how the interpretation of the study’s findings may be affected by the study limitations. In the final bullet point of ‘What Do These Findings Mean?’, please describe the main limitations of the study in non-technical language.

The author Summary should immediately follow the Abstract in your revised manuscript. This text is subject to editorial change and should be distinct from the scientific abstract. Please see our author guidelines for more information: https://journals.plos.org/plosmedicine/s/revising-your-manuscript#loc-author-summary

**Authors:** We added the Author Summary immediately following the Abstract. In Page 4-5, we wrote:

*“****Why Was This Study Done?***

- *Using inappropriate controls in randomized trials of new cancer drugs can expose patients to harm and produce potentially unreliable results of clinical efficacy.*
- *In November 2021, China’s Center for Drug Evaluation issued the Guidance on Clinical Value-Oriented Oncology Drug Research and Development. It stated that an active control arm should be selected with consideration of whether it reflects the optimal treatment in current clinical practices.*
- *Previous descriptive analysis showed that oncology trials are expanding in China, however, no literature evaluated the quality of their control arms and the number of participants at risk of exposure to suboptimal treatments in China.*

***What Did the Researchers Do and Find?***

- *This observational study reports the annual trend and proportion of 453 Phase-II/III and -III randomized oncology trials registered in China between 2016 and 2021 that adopted suboptimal control arms. We also calculated the cumulative number of trial participants at risk of exposure to suboptimal treatments.*
- *We observed a fluctuating yet overall increasing trend in the number of trials and participants that used suboptimal comparators during our observation period. Overall, 60 trials adopted a suboptimal control arm and the cumulative number of trial participants at risk of exposure to suboptimal treatments totaled 18,610 by the end of 2021.*
- *Trials with a suboptimal control reported higher but statistically non-significant proportions of positive results than those using an optimal control.*

***What Do These Findings Mean?***

- *Trial sponsors, ethical review boards, and oncologists should make collaborative efforts to protect patients from unnecessary harm and drugs with uncertain clinical benefits over the existing standard of care. Regulatory agencies should be cautious when reviewing investigational new drug applications whose supporting trial used a suboptimal control and when making subsequent market authorization decisions.*
- *A limitation of this study is the reliance on clinical guidelines that could vary across cancer types and time in assessing the quality of the control groups. Hence, though oncologists and clinical pharmacists were consulted when assessing the sample trials, our results may have inherent subjectivity.”*

**INTRODUCTION**

Please indicate whether your study is novel and how you determined that.

**Authors:** Thanks for this important point. We emphasized the novelty statement in the *Introduction* section as suggested. In Page 7 Line 132, we wrote:

*“There is no empirical evidence, however, about the appropriateness of the control arm of these cancer drug clinical trials. Moreover, the association between the quality of the control arm and trial results was rarely scrutinized. In this observational study, we aim to identify randomized oncology registration trials using a suboptimal control arm in China and estimate the number of trial participants at risk of receiving suboptimal treatments. For RCTs with published results, we further investigated the proportion of trials with statistically significant results on their primary endpoint and examined the association between the adoption of a suboptimal control arm and concluding statistical significance.”*

If there has been a systematic review of the evidence related to your study (or you have conducted one), please refer to and reference that review and indicate whether it supports the need for your study.

**Authors:** Thank you for this comment. We searched PubMed and Google Scholar for original studies using the search terms “control arm”, “suboptimal”, “randomized trials”, and “cancer”. Several observational studies conducted in the US investigated the quality of control groups in randomized trials of the Food and Drug Administration-approved cancer drugs or oncology trials published in 11 top journals in the last three years. We have added references to several previous studies in the *Introduction* section. In Page 6 Line 104 and Page 7 Line 122, we wrote:

*“[P]revious research has shown that 17% (16/97) of cancer drug approvals of the US Food and Drug Administration (FDA) between 2013-2018 were supported by RCTs that used suboptimal controls.[4] That is, the control agent had not been recommended by a relevant guideline or had been demonstrated to be inferior to available therapy. Similarly, seven of 49 (14%) RCTs investigating drugs for multiple myeloma in the US randomized participants to control arms despite the availability of alternative regimens proven to have superior clinical efficacy.[5] More recently, there has been a growing concern about the selection of inappropriate control arms in trials of metastatic castrate-resistant prostate cancer[6, 7] and hematological malignancies.[8-10]”*

*“Using suboptimal comparators in RCTs, especially unlicensed agents or regimens, can expose patients to unnecessary substandard therapy and produce unconvincing comparative efficacy results of new cancer drugs over the existing standard of care.[15] Of the 387 oncology RCTs published in 11 major journals between 2017 and 2021, 43 (11%) were deemed to have a suboptimal control, with a total of 9,505 patients assigned to the suboptimal treatment arms.[16]”*

Line 51 – ‘14% of RCTs…’ how many was this? It might be helpful to also include the approximate number of participants that these trials included (if known) such that the reader can get a clear picture of the extent of the issue.

**Authors:** We added the specific number of trials reported in the literature. In Page 6 Line 107, we wrote:

*“[S]even of 49 (14%) RCTs investigating drugs for multiple myeloma in the US randomized participants to control arms despite the availability of alternative regimens proven to have superior clinical efficacy.[5]”*

Since Ref#5 did not report the number of participants in its sample trials, we performed an updated literature review and added a recently published study in the *Introduction* section. In Page 7 Line 122, we wrote:

*“Of the 387 oncology RCTs published in 11 major journals between 2017 and 2021, 43 (11%) were deemed to have a suboptimal control, with a total of 9,505 patients assigned to the suboptimal treatment arms.[16]”*

Additional detail on how control arms are determined and by whom would be helpful. Does the problem exist at the level of the pharmaceutical company, or the regulators or both?

**Authors:** Thanks for this advice. We believe trial sponsors shoulder the primary responsibility in the selection of the control group. This was added in the *Introduction* section as suggested. In Page 6 Line 100, we added:

*“When designing an RCT, sponsors or investigators choose control groups prudently since they affect the inferences that can be drawn from the trial, the ethical acceptability of the trial, and the acceptability of the results by regulatory authorities.[3]”*

As also emphasized by Reviewer#1, IRBs are in charge of protecting patients from unnecessary suboptimal treatment. We also added this on Page 7 Line 124:

*“At the core of considerations in an ethical review are principles of respect for persons, beneficence (do not harm and maximize possible benefits and minimize possible harms), and justice,[18] hence, research resources could be more effectively utilized if institutional review boards (IRBs) embrace their responsibilities by ensuring that each trial receives meaningful scientific review.[19]”*

**METHODS and RESULTS**

We agree with the statistical reviewer, please see below, that investigation as to whether use of a sub-optimal control arm was associated with higher chances of concluding statistical significance would strengthen the manuscript.

**Authors:** Thank you and the Reviewers. To address this comment, which was also stated by Prof. Kimmelman and Prof. Wason, we further investigated the association between the use of suboptimal trial control and concluding statistical significance in the revised manuscript.

Our stepwise search via PubMed found that 105 sample trials (23.2%) have publicly available results on their primary endpoint. Trials with suboptimal control reported higher but statistically non-significant proportions of positive results than those used optimal control (100.0% vs 83.9%, *p*=0.208). We added these to the revised manuscript (*Abstract*, Line 58; *Identification of Publication of Sample Trials* in the *Methods* section, Line 205; *Availability and statistical significance* of trial results, in the *Results* section, Line 284).

Please ensure that percentages are quantified with numerators and denominators, not just percentages.

**Authors:** Thanks. We added numerators and denominators of all reported percentages as suggested.

Where 95% CIs are reported, please also report values. Please report as p <0.001 and where higher as p= [exact p value]

**Authors:** We reported values together with 95% Cis. P-value were reported as p <0.001 or exact p value throughout the revised *Methods* and *Results* section. Please check.

Please separate upper and lower bounds of CIs with commas as opposed to hyphens as these can be confused with reporting of negative values.

**Authors:** We separated the upper and lower bounds of CIs with commas in the *Results* section and Tables as suggested.

**DISCUSSION**

Thank you for structuring your discussion according to PLOS Medicine’s style. Please remove all sub-headings from the discussion such that it reads as continuous prose.

**Authors:** We removed all sub-headings from the *Discussion* section as required.

**TABLES**

Table 1 – thank you for indicating that your analyses are adjusted. Please explicitly detail which factors are adjusted for in the caption. To help facilitate transparent data reporting, please also include the unadjusted analyses for comparison. Please report p as <0.001 and where higher the exact p value. Please define ‘CI’ for the reader (either the column header or the footnote). Please separate upper and lower bounds of 95% CIs with commas as opposed to hyphens as these can be confused with reporting of negative values.

**Authors:** Thanks. We added the unadjusted odds ratio and the adjusted factors in Table 1 and its caption as also suggested by Reviewer#1. In Page 29, we wrote:

*“^c^ Adjusted ORs were obtained from the multivariable logistic regression model. Factors identified in the univariable analysis were adjusted: year of ethical approval, recruitment region, line of therapy, and cancer site.”*

We also added the univariable logistic regression in the *Data Analysis* section. In Page 12, Line 233, we wrote:

*“To identify potential predictors of the use of suboptimal control, the univariable logistic regression, Chi-square test, and Fisher’s exact test were performed to test differences between groups in categorical variables.”*

All p-values and 95% confidence intervals were modified as required. Abbreviations were added in the Table caption.

Table 2 – please either define ‘No.’ or replace with ‘number’.

**Authors:** We replaced “No.” with “number” in Tables as suggested.

**FIGURES**

Figure 2 – please define ‘No.’ or use the word ‘number’.

**Authors:** We replaced “No.” with “number” as suggested.

**DECLARATION STATEMENTS**

Lines 305, 306 & 312 – please remove these statements and include only in the manuscript submission form when you re-submit the manuscript. In the event of publication these will be compiled as metadata.

**Authors:** Thanks. We removed these statements as suggested.

**REFERENCES**

For in-text reference callouts please place citations in square brackets and preceding punctuation. For example, [1,3,6].’ Please check and amend throughout all subsections of the manuscript including the supporting information as relevant.

In the bibliography, please list up to but no more than 6 author names followed by et al.

Please ensure any web references include an ‘Accessed [date]’

Journal name abbreviations should be those listed in the National Center for Biotechnology Information (NCBI) databases.

Please see our website for other reference guidelines https://journals.plos.org/plosmedicine/s/submission-guidelines#loc-references

**Authors:** Thank you very much for this helpful advice. We downloaded the EndNote Style and replaced the citations throughout our manuscript.

**SUPPORTING INFORMATION**

Please cite your Supporting Information as outlined here: https://journals.plos.org/plosmedicine/s/supporting-information

In the published article, supporting information files are accessed only through a hyperlink attached to the captions. For this reason, you must list captions at the end of your manuscript file. You may include a caption within the supporting information file itself, as long as that caption is also provided in the manuscript file. Do not submit a separate caption file.

**Authors:** Thanks. We listed *Supporting Information* captions at the end of the manuscript file (Page 34: Supporting information captions).

eTable 2 and 3 – abbreviations for information detailed in the ‘Target’ column are not defined in the footnote (HER-2, for example). Please include definitions for all abbreviations.

**Authors:** Sorry for the omission. We checked throughout the *Supporting Information* and added definitions for all abbreviations.

**COMMENTS FROM THE REVIEWERS:**

**Reviewer #1 (Jonathan Kimmelman, McGill University):** In this paper, authors assess the proportion of cancer RCTs in China that use suboptimal comparators by conducting a systematic search for RCTs using Chinese databases, and then assessing comparator against contemporaneous clinical practice guidelines (CPGs) in China. From a (saturation) sample of 454 trials, they find that about 14% of RCTs used suboptimal comparators. Trials recruiting only in China, as well as adjuvant and maintenance trials, were at elevated risk of using suboptimal comparators.

With some of the provisos laid out below I think this is an excellent manuscript addressing an important question. Why do trials if they use an inappropriate control? In my opinion the major cancer journals do not seem to want to answer this question (or reckon with the fact that this occurs). The manuscript is also very well researched and embeds the study in the a growing literature on comparator choice in cancer trials. Third, the study uses what are now considered well- established methods for assessing comparator quality. What makes this study unique and IMO potentially impactful is its focus on China- a major center for biomedical research- it is hard for those of us living in North America or Europe to get a good read on research practices in China. Also, the paper is written very clearly, and the results are transparently reported (with a good Supplementary Materials).

**Authors:** We truly appreciate these positive comments.

I offer the below comments and suggestions in the spirit of helping the authors refine the presentation and analysis.

- line 57- point about external validity is incorrect. The effect is still externally valid - comparative effects can be generalized to other settings. The problem is that the generalization is irrelevant since the comparison in the trial does not reflect current standards. Suggest rewording.

**Authors:** Thank you very much for this correction. We modified our expression in the revised manuscript as suggested. In Page 6, Line 113, we wrote:

*“Adopting a suboptimal control group in a cancer drug registration RCT may bias its results in favor of the experimental arm and compromise its probability of informativeness in evolving treatment landscape as the comparator does not reflect the current standard of care.”*

- Around line 57-65. I strongly feel that authors should appeal to basic ethical principles, including those expressed in Declaration of Helsinki or elsewhere, that say that patients should be provided best available therapy in comparator arms. Better yet would be an appeal to the principle of clinical equipoise, which is clearly violated when suboptimal comparators are used. In discussion, authors invoke ethical standards and Helsinki. But my feeling is that this should come earlier. It is simply unethical to randomize patients with life threatening diseases to suboptimal treatments, full stop. Any occurance of that means IRBs are not doing their job. Nor regulators. Nor physicians who agree to recruit their patients.

**Authors:** Thank you for this comment. We revised the second paragraph in the *Introduction* section as suggested. In Page 7, Line 124, we wrote:

*“At the core of considerations in an ethical review are principles of respect for persons, beneficence (do not harm and maximize possible benefits and minimize possible harms), and justice,[18] hence, research resources could be more effectively utilized if institutional review boards (IRBs) embrace their responsibilities by ensuring that each trial receives meaningful scientific review.[19]”*

- Around line 99- why did authors only include adult trials? What proportion enrolled both adults and children?

**Authors:** Thank you for this comment. There were two considerations behind us including only adult trials. First, only four registered phase-III childhood cancer trials were eligible for this study (CTR20192678, CTR20192718, CTR20200241, and CTR20212641). The limited sample size would restrict the feasibility of separating pediatric trials into a subgroup. More importantly, the availability of childhood cancer guidelines is poor in China.^[1]^ The Chinese Society of Clinical Oncology (CSCO) did not issue its first guideline for children- *CSCO Guideline for Lymphoid Malignancies in Children and Adolescents*- until 2020 and thus we were unable to assess the control arm of the four trials (that were registered before the issue of the concerned guideline in China).

We revised the Flowchart (Fig 1) to give more information on the number of pediatric trials identified in our search. We also added this in the *Limitation* section. In Page 20, Line 417, we wrote:

*“Eighth, we only included trials conducted among adults due to the limited number of phase-3 childhood cancer trials (only four) and the poor availability of clinical guidelines concerned with childhood cancer in China before 2021.”*

- line 188-128- cross referencing NCCN and FDA approvals is a really nice methodological addition here.

**Authors:** Thank you very much.

- line 130: were extractions performed in duplicate and independently for eligibility and guideline matching? If so, what was the agreement rate? If not, can authors substantiate that there was enough agreement as to assure the reader of accuracy and reproducibility of extrations / matching?

**Authors:** We apologize for this unclear expression (i.e., *“Three investigators (Y. Z., D.C., and Z.L.) extracted the indication and eligibility criteria of all RCTs”*). Data extraction and guideline matching were performed independently. We also checked the raw data and worksheet to calculate the agreement rate (94.9%, n=430/453). In Page 10, Line 198, we wrote:

*“Two investigators (D.C. and Z.L.) independently extracted information about the indication and eligibility criteria of all RCTs and matched corresponding guideline recommendations for further investigation. The initial agreement rate achieved 94.9% (n=430/453). Another investigator (Y.Z.) resolved disagreements.”*

- I am a little confused. I presume some trials in the sample were multicentric and international. So when counting numbers of patients, how did the authors deal with the fact that some patients may have originated from outside China (where standard of care might have varied or been deemed adequate)?

**Authors:** Thank you for this comment, and we are pleased to provide further clarification. In China’s National Medical Products Administration Registration and Information Disclosure Platform for Drug Clinical Studies, the number of trial participants recruited from domestic and foreign countries was reported separately. We translated a screenshot of this section for your kind perusal.

In this study, we calculated the number of domestic enrollees of sample RCTs with a suboptimal control arm. In Page 11, Line 224, we wrote:

*“We calculated the annual and cumulative number of planned enrollees of sample RCTs with a suboptimal control arm in mainland China. For trials that did not disclose their planned sample size, we relied on the actual number of participants enrolled as of 31 August 2022.”*

- Was this study prespecified in a protocol, with all hypotheses prespecified? If so, authors should name the database (e.g. Center for Open Science) and provide the registration record. If not, author should state that the study protocol and analyses were not pre-specified. Prespecification is increasingly the norm in meta-research of this type, though this reviewer acknowledges that many authors routinely bypass this fairly simple quality control measure.

**Authors:** Thank you for this kind reminder. We apologize for not registering for a research protocol. In further studies, we will register our research with a pre-specified analysis plan. We added this in the *Limitation* section as suggested. In Page 20, Line 420, we wrote:

*“Lastly, the study protocol and analyses were not pre-specified in an open database.”*

- For optimal treatments, did authors assess dose or schedule of comparator arms? Sometimes studies use appropriate comparators but at a lowered dose, or on an inappropriate schedule. If authors did not do this, this should be stated as a limitation. Or better yet, author may want to assess this in their 86% of trials that used optimal compartors- or at least they might want to do this in a sample to see if it occurred.

**Authors:** We excluded four dose-optimization trials (marked in Figure 1), given some of them had not received market authorization and consequently we could not determine the “optimal dose or schedule”. Besides, some of the post-market dose optimizations may be appropriate due to the quality of design of the pre-approval dose-finding trial.^[2]^ We added this as a limitation as suggested. In Page 20, Line 412, we wrote:

*“Seventh, we only evaluated the quality of the control arm of RCTs and thus did not analyze the potentially inappropriate use of single-arm designs for conditions with existing standards of care. With the increasing number of non-randomized oncology trials in China, further attention should be given to the rationality of using single-arm trials as the pivotal evidence for regulatory approval. Similarly, we only assessed the regimen rather than the dose or schedule of comparator arms. Further study may be necessary to evaluate the quality of the latter if a standard dose or schedule is built.”*

- Line 181-3. This is tricky. With respect to category 4 of Box 1: Suppose two drugs are recommended, but one (A) is recommended at a higher level of evidence than the other (B). Would it not be the case that, if (B) is associated with less toxicity or easier administration (e.g. oral instead of IV), there might be sound reasons to recommend B rather than A (hence B IS a standard of care choice)? For combination- did authors check for simple substitutes of similar drugs, in which case while the exact combo is not the same, there are good reasons to think of the combo as equivalent to the one listed in the guidelines? [to some extent this is addressed around line 226 but I suspect this could be regarded as a controversial point]

**Authors:** Thank you for this thoughtful comment. We gave this issue a second thought and reassessed the two trials applicable to Category 4 carefully. We reviewed the supporting literature for CTR20191073 (the trial that investigated camrelizumab vs. FOLFOX4 [Level of Evidence: 2] to treat advanced untreated hepatocellular carcinoma while the guideline recommends targeted therapy [sorafenib, Level of Evidence: 1]) and CTR20190416 (the trial that investigated zanubrutinib vs. bendamustine± rituximab [Level of Evidence: 2A] to treat untreated chronic lymphocytic leukemia, while the guideline recommends ibrutinib [Level of Evidence: 1]) and screened MEDLINE to find whether a superior efficacy had been demonstrated in a prior RCT.

For advanced untreated hepatocellular carcinoma, no direct comparison between sorafenib and FOLFOX4 had been made before the trial started (or even by now).^[3]^ ^[4]^ For untreated chronic lymphocytic leukemia, the A041202 Study (NCT01886872) published before the IRB approval time of CTR20190416 showed no significant difference among ibrutinib and bendamustine plus rituximab with regard to overall survival, making bendamustine plus rituximab a clinically appropriate control.^[5]^ Therefore, we removed the previous criterion for Category 4 in the revised *Methods* and *Results* section.

- Totally up to authors, but they may want to cite (and link in to) my work on uninformative trials- in particular Zarin et al (JAMA 2019) and Hutchinson et al (eLife 2022).

**Authors:** Thanks for this helpful literature. We cited them in the revised *Introduction* section. In Page 6, Line 115 and Page 7, Line 126, we wrote:

*“Information from new cancer drug trials with an inferior control group would confound regulatory or clinical decision-making by regulators and oncologists. (Nat Rev Clin Oncol. 2022;19(7):425-6;* ***Elife. 2022;11:e79491****.)”*

*“[R]esearch resources could be more effectively utilized if institutional review boards (IRBs) embrace their responsibilities by ensuring each trial receives meaningful scientific review. (****JAMA. 2019 Sep 3;322(9):813-814****.)”*

- It's interesting that comparator choice seems more problematic with adjuvant and maintenance trials. Do authors have any thoughts on why this might be so? Might this relate to the point I made above about safety (a safer drug, taken over a more prolonged period but supported on weaker evidence, might be preferable to a more toxic drug supported by strong evidence).

**Authors:** We examined the results and found that the principal drivers of an adjuvant and maintenance trial to be assessed as “suboptimal” were hepatocellular carcinoma and lung cancer, and ovarian cancer, separately. These were further interpreted in the Discussion section. In Page 18 Line 365, we wrote:

*“These findings could be explained by the evolving treatment landscape and discrepancies between the landscape in China and in other regions principally. For high-risk Stage IB NSCLS, China’s latest clinical practical guideline did not recommend adjuvant chemotherapy due to the absence of high-level evidence while the NCCN Guideline recommended observation or adjuvant chemotherapy/osimertinib (for EGFR exon 19 deletion or L858R). For hepatocellular carcinoma, adjuvant transarterial chemoembolization after curative resection got a Grade-I recommendation in China’s guideline (Level-of-evidence: 2A) with documented survival gain[41] while there was no global consensus on the treatment being a standard of care. For BRCA1/BRCA2 mutation-positive platinum-sensitive epithelial ovarian cancer, though poly ADP-ribose polymerase inhibitor (PARPi) has been recommended as the maintenance therapy since 2019, still, several placebo-controlled trials on next-in-class PARPi have been initiated subsequently.”*

- For readers unfamiliar with Chinese clinical practice guidelines, can authors say something about the quality of Chinese CPGs? For example, are there formal procedures used to evaluate and prioritize drugs for recommendations? Are they supported by systematic reviews? The strength of claims in this paper rests on the credibility of CPGs, so a comment (and evidence) on this would be very helpful.

**Authors:** Thank you for this comment, we are pleased to provide further clarification. We mainly refer to CSCO Guidelines to assess the quality of control arms. The development of CSCO Guidelines follows the level of evidence and consults expert consensus.^[6]^ We listed the detailed information below for your kind perusal.

Besides, for some indications, CSCO also developed evidence-based recommendations in cooperation with the American Society of Clinical Oncology,^[7]^ which convened an Expert Panel to conduct a systematic review of relevant studies and develop recommendations for clinical practice.^[8]^ These methodologies of guideline development safeguard the guidelines’ quality and relevance to the Chinese population.

To make it clear to the audience, we also added a description about the methodology of CSCO’s guideline development in the *Methods* section as suggested. In Page 8, Line 155, we wrote:

*“The development of CSCO Guidelines follows the level of evidence and expert consensus. CSCO categorizes evidence-based recommendations into Grade I (Evidence level 1A and some Evidence level 2A), II (Evidence level 1B and some Evidence level 2A), and III (Evidence level 2B and 3).”*

For cancer types without a specific CSCO guideline, we searched clinical practice guidelines published by other academic societies, including subcommittees of the Chinese Medical Association and the Chinese Anti-Cancer Association. These societies established nationwide specialist panels to consolidate medical evidence for specific cancers.^[9]^ Although we believe these academic societies approach developing guidelines in a rigorous manner and produce guidelines that can be deemed as the most reliable ones in China, due to the absence of their methodology appraisal, the development and update of these guidelines may have flaws that are unknown to us. This was also clarified in the *Limitation* section. In Page 19, Line 397, we wrote:

*“Third, some guidelines may be subject to flaws, such that they were not updated timely or they expanded to include some options which were actually not relevant to the population being studied. Although we used FDA approvals and NCCN recommendations to re-evaluate the quality of control arm of multinational trials, clinical benefits and level of evidence supporting their approval and recommendation may have discrepancies and deficiencies. To mitigate these impacts, we invited several oncologists and clinical pharmacists to cross-check if our results were in line with their observations in actual clinical practices.”*

- Beyond the scope of the article, but it would be great (and pretty easy, I think) to assess the proportion of trials that are positive on their primary outcome in the standard of care vs. suboptimal standard of care strata. A finding of a difference would underscore the impact of comparator bias. A null result would provide somewhat reassuring evidence that patients in comparator arms have not been harmed (though, of course, there is always the possibility that companies pursuing less promising drugs are more prone to picking weaker comparators to give their less promising drugs a boost).

**Authors:** Thank you for this constructive advice. To address this comment, which was also stated by the Editor and Reviewer #2, we added an *Identification of Publication of Sample Trials* in the *Methods* section. In Page 10, Line 205, we wrote:

*“For each trial, we used a stepwise approach to systematically search for peer-reviewed publications of its results, given the results submitted to the NMPA Registration and Information Disclosure Platform for Drug Clinical Studies are not publicly available. First, we searched ClinicalTrials.gov (the US National Library of Medicine database of clinical trials) using Study IDs (or Study Title/ Acronym when necessary) to match their National Clinical Trial (NCT) number. Second, we searched PubMed using the NCT number and reviewed all corresponding publications. If no publication was found through these two steps, we further searched PubMed for RCTs using a precision-maximizing search strategy and a combination of terms including the drug’s name (if unavailable, drug code) and indication.[22]*

*We reviewed the titles and abstracts of all records to identify the primary publications reporting trial results. Among all eligible publications, we extracted information about a trial’s primary endpoints.* *If more than one publication reported trial results, we relied on the initial one that reported the preplanned analysis of the primary endpoint(s). We also examined the availability of a pre-specific statistically significant benefit of the sample RCT. For trials set coprimary endpoints, the results were deemed as statistically significant if the difference between the experimental and the control group was considered to be statistically significant for at least one endpoint.”*

We also added *Availability and statistical significance of trial results* subsections in the *Results* section. The results showed that there was no statistically significant association between the use of a suboptimal control and concluding statistical significance on the primary endpoint. In Page 14, Line 284, we wrote:

*“Of 1,132 publications obtained from MEDLINE by September 2023, we identified 131 reporting the preplanned results of 105 sample trials (23.2%). Among them, 105 reporting the initial trial results were included for data extraction. Trials mostly used progression-free survival (56/105, 53.3%) and overall survival (24/105, 22.9%) as the primary endpoint. Some studies (14/105, 13.3%) used overall survival and progression-free survival concomitantly as the primary endpoints (see S1 Table 4). Of the 93 trials that were deemed to have used an optimal or potentially optimal control, 78 (83.9%) reported statistically significant results while 15 (16.1%) concluded statistical non-significance (Table 3, see S1 Table 5 for details). Of the trials that were deemed as having a suboptimal control, all (12/12, 100.0%) reported statistically significant results. No statistically significant association was observed between the use of a suboptimal control and concluding statistical significance on the primary endpoint (p=0.208).”*

We further interpreted this in the *Discussion* sections. In Page 16, Line 330, we wrote:

*“Since large-scale multicenter oncology trials are costly and entail a high risk of failure, trial sponsors are not always incentivized to conduct head-to-head studies of their product with the best-proven therapy.[5] Our results also showed that compared with trials with optimal control, those who adopted a suboptimal control had a higher proportion of concluding positive results, which was in favor of the pharmaceutical companies, though this was not statistically significant.”*

This finding might be biased due to the short follow-up of trial results and publication bias. This was also acknowledged in the *Limitation* section. In Page 20, Line 410, we wrote:

*“Sixth, publicly available data may represent a selective subset of trial information since only 23.2% of sample trial results were published by the end of our observation.”*

- Also beyond the scope of the article: for multinational studies, authors consulted NCCN and FDA approvals. This affords authors to ask an additional question: to what extent were multinational trials enrolling in China prone to using comparators that were below standard of care in the U.S.? One would a priori predict that multinational studies enrolling in both U.S. and China would be "enriched" for use of suboptimal N American comparators, since such trials might under-accrue in the U.S. but more vigorously accrue in China. Totally optional (authors may want to see Awan et al Annals of Internal Medicine 2022 for a discussion of U.S. multinational cancer trials that also recruit in middle income countries).

**Authors:** We fully agree with your comments. However, the absence of an internationally harmonized standard of care, mainly driven by the unequal accessibility to some novel therapies, made it difficult to assess the quality of all RCTs registered in China against the US standard of care. To address this issue as much as possible, in this study, if a multinational trial encompassed a specific indication on which a treatment consensus had not been reached in China, we reviewed whether there existed a standard of care in the former National Comprehensive Cancer Network (NCCN) Guidelines, which have a worldwide influence on clinical practice, at that time (Page 8, Line 189).

We appreciate the Reviewer for providing a potential explanation referred to as “trial recruitment bias” and we added this to the *Discussion* section. In Page 17, Line 344, we wrote:

*“Compared with international multicenter RCTs, trials that recruited participants only in China seemed more likely to adopt an inferior control. A potential explanation is that trials initiated by multinational companies are more likely to recruit participants in high-income countries and thus the proportion of their trials using a suboptimal control might be underestimated.”*

- Also I wondered whether authors considered other subgroup analyses, including whether drug was combo vs. mono, whether company was large vs. small (biotech).

**Authors:** Thank you for this advice. We added the type of therapy (combo vs. mono vs. combo/mono [three-arm design]) as suggested. The univariable analysis showed a statistically non-significant association between therapy type and the use of a suboptimal control (*p*=0. 0.403, Table 1).

As for the scale of pharmaceutical companies, we apologize that we were unable to categorize them into large vs. small companies since some of them are not listed companies in China, making the company scale not publicly available. To address this issue as much as possible, we compared the applicant type (China domestic vs. Multinational company) in the subgroup analysis (*p*=0.183, Table 1).

- In abstract and elsewhere (e.g. line 190), authors should report the cumulative number of patients at risk of entering a trial with suboptimal comparators in terms of proportions, not just absolute numbers.

**Authors:** We added the proportion of patients at risk of being randomized to a suboptimal treatment as suggested. In Page 3, Line 52, we wrote:

*“The cumulative number of trial participants at risk of exposure to suboptimal treatments totaled 18,610 by the end of 2021, contributing 15.1% to the total number of enrollees of all sampled RCTs in this study.”*

- Fig 2, panel B. Please use other colours for bars. Very hard to distinguish these colours for many readers who are colour blind.

**Authors:** Sorry for this inconvenience. Following your kind advice, we replaced Fig 2 with grayscale only.

Very minor suggestions:

- Small grammar errors at line 188

**Authors:** Thanks. We modified our expression. In Page 14 Line 278, we wrote:

*“The annual number of participants who would be assigned to receive suboptimal treatments in China increased over time.”*

- Line 195- report as p < some value (e.g. 001)

**Authors:** Thanks. We reported the p-value as p <0.001 or p=[exact p value] where the p-value was no smaller than 0.001 throughout the manuscript, as also suggested by the Editor.

An additional thought- not necessary to address. The finding of 14% is pretty much in line with proportions reported using U.S. based or European trial samples. I think an additional insight this paper provides is that Chinese trials are no more misaligned with contemporary ethical standards than are U.S. or European studies- that is reassuring in a relative sense but discouraging in an absolute sense.

**Authors:** Thank you very much. We fully agree and added this statement in the *Discussion* section. In Page 16, Line 324, we wrote:

*“Although it is challenging to compare regulatory outcomes across different settings and time periods, it seems that the proportion of RCTs registered in China with an optimal control was comparable to those in other developed countries, which suggests that trials registered in China are no less aligned with contemporary ethical standards than are in the U.S. or European countries.”*

In short: timely study addressing an important question. Findings are important, work is original, methods are appropriate and transparent. I think there are things authors can do to improve the presentation, and they can report a bit more information on methodology so the reader can get a sense of quality checks in extraction and analysis. But all in all, this manuscript is promising. Apologies to authors (and editors) if there is anything crucial that I missed.

**Authors:** Thank you very much again for these insightful and helpful comments above. In the revised manuscript, we have tried our best to address them. We believe these changes have strengthened our paper. Please find enclosed our revised manuscript.

**Reviewer #2: Statistical review (James Wason)**

This paper investigates the proportion of phase III cancer trials run in China that used a sub-optimal control arm (i.e. one that did not match treatment guidelines). The paper provides an interesting analysis. It uses (appropriately) straightforward analyses so there wasn't too much for me to comment on.

1. Page 5 - can the authors say more about the timing of when an arm was sub-optimal. If a trial started in (for example) 2017 using a control that was recommended in 2016 but not 2017 would that be classified as suboptimal? If standard of care changes during the course of the trial but the control arm is not updated, is the trial classified as using a suboptimal control arm?

**Authors:** Thank you very much for pointing out this important issue and we apologize for the ambiguous expression. We referred to guidelines published before the ethical approval date to assess the quality of the control group, under the principal consideration that trial sponsors and institutional review boards might need time to get access to the latest published guidelines. Control arms became outdated during the course of trials due to a change in the standard of care was not deemed as suboptimal, in line with previous studies.^[10]^^[11]^ We further clarified this in the *Methods* section to make this clear to the audience. In Page 9 Line 177, we wrote:

*“For each trial in our sample, we defined the quality of the control arm as suboptimal if: (1) the control was not recommended by the concerned* *clinical practice guideline for a specific indication issued prior to the trial’s IRB approval (i.e., different from the regimen recommended in guidelines published before the trial’s IRB approval); (2) the control drug or regimen was specified in the guideline but not recommended (e.g., the control arm was a single agent when the guideline published before the trial’s IRB approval recommended doublet therapy); or (3) the control arm was among the recommended list of therapies but there existed a drug (or drugs) with a higher level of recommendation.”*

2. Page 7 - can the authors provide the list of covariates considered in the model? Were these pre-specified?

**Authors:** Thank you for this comment and we are pleased to provide further clarification. To identify the potential predictors of the use of suboptimal control, the univariable logistic regression, Chi-square test, and Fisher’s exact test were performed to test differences between groups. Factors found to be significant in univariable analysis were adjusted in the multivariable logistic regression model. We added the adjusted factors in the Table 1 caption as also suggested by the Editor. In Page 29, we wrote:

*“****^b^*** *p-values were obtained from the univariable logistic regression, Chi-square test, and Fisher’s exact test.*

***^c^*** *Adjusted ORs were obtained from the multivariable logistic regression model. Factors identified in univariable analysis were adjusted: year of ethical approval, recruitment region, line of therapy, and cancer site.”*

3. Page 9 (and tables) 'p= 0.000' - I would report this as p<0.001 (but check PLOS guidance on p-values to make sure).

**Authors:** Thanks. We reported p values as <0.001 or the exact p-value where it was no smaller than 0.001, as also required by the PLOS Guidance.

4. I'm not sure if the authors extracted information on whether the trial found a statistically significant result for the primary outcome. If so I would have found it interesting to see whether use of a sub-optimal control arm was associated with higher chances of concluding statistical significance.

**Authors:** Thank you for this constructive advice. To address this comment also recommended by the Editor and Reviewer #1, we added an *Identification of Publication of Sample Trials* in the *Methods* section. In Page 10, Line 205, we wrote:

*“For each trial, we used a stepwise approach to systematically search for peer-reviewed publications of its results,[32] given the results submitted to the NMPA Registration and Information Disclosure Platform for Drug Clinical Studies are not publicly available. First, we searched ClinicalTrials.gov (the US National Library of Medicine database of clinical trials) using Study IDs (or Study Title/Acronym when necessary) to match their National Clinical Trial (NCT) number. Second, we searched MEDLINE (via PubMed) using the NCT number and reviewed all corresponding publications. If no publication was found through these two steps, we further searched PubMed for RCTs using a precision-maximizing search strategy and a combination of terms including the drug’s name (if unavailable, drug code) and indication.[22]*

*We reviewed the titles and abstracts of all records to identify the primary publications reporting trial results. Among all eligible publications, we extracted information about a trial’s primary endpoints (i.e., statistical analysis plan, trial results, and statistical significance). If more than one publication reported trial results, we relied on the initial one that reported the preplanned analysis of the primary endpoint(s). We also examined the availability of a pre-specific statistically significant benefit of the sample RCT. For trials set coprimary endpoints, the results were deemed as statistically significant if the difference between the experimental and the control group was considered to be statistically significant for at least one endpoint.”*

We also added *Availability and statistical significance of trial results* subsections in the *Results* section. The results showed that there was no statistically significant association between the use of suboptimal control and concluding statistical significance on the primary endpoint. In Page 14, Line 284, we wrote:

*“Of 1,132 publications obtained from MEDLINE by September 2023, we identified 131 reporting the preplanned results of 105 sample trials (23.2%). Among them, 105 reporting the initial trial results were included for data extraction. Trials mostly used progression-free survival (56/105, 53.3%) and overall survival (24/105, 22.9%) as the primary endpoint. Some studies (14/105, 13.3%) used overall survival and progression-free survival concomitantly as the primary endpoints (see S1 Table 4). Of the 93 trials that were deemed to have used an optimal or potentially optimal control, 78 (83.9%) reported statistically significant results while 15 (16.1%) concluded statistical non-significance (Table 3, see S1 Table 5 for details). Of the trials that were deemed as having suboptimal control, all (12/12, 100.0%) reported statistically significant results. No statistically significant association was observed between the use of suboptimal control and concluding statistical significance on the primary endpoint (p=0.208).”*

We further interpreted this in the *Discussion* sections. In Page 16, Line 330, we wrote:

*“Since large-scale multicenter oncology trials are costly and entail a high risk of failure, trial sponsors are not always incentivized to conduct head-to-head studies of their product with the best-proven therapy.[5] Our results also showed that compared with trials with optimal control, those who adopted a suboptimal control had a higher proportion of concluding positive results, which was in favor of the pharmaceutical companies, though this was not statistically significant.”*

This finding might be biased due to the short follow-up of trial results and publication bias. This was also acknowledged in the *Limitation* section. In Page 20, Line 410, we wrote:

*“Sixth, publicly available data may represent a selective subset of trial information since only 23.2% of sample trial results were published by the end of our observation.”*

**Reviewer #3:** The authors present a retrospective, observational study to investigate the proportion of randomized trials of investigational cancer drugs that used a suboptimal control arm and the number of trial participants at risk of exposure to suboptimal treatments in China. These findings carry significant policy and clinical implications, as the use of a suboptimal control arm can hinder therapeutic innovation and compromise the benefits for participants. Overall, the methodology is sound and the manuscript is well-written. However, I have a few concerns that the authors should address before making definitive conclusions.

1.Some of the "suboptimal" control arms identified by the authors are questionable (eTable 3). For example, in the case of CTR20171559, it was actually conducted in the ≥3rd-line setting for treating advanced colorectal cancer, not the 2nd-line setting, making "FOLFOX/FOLFIRI/CapeOx±Cetuximab/Bevacizumab" not the guideline-recommended regimens in this context.

**Authors:** Thank you very much for this detailed comment, we are pleased to provide further clarification. We further discussed this with our co-author Dr. Siyuan Cheng, who is a gastrointestinal oncologist, and reached a consensus that this trial was indicated for a 3rd- or later-line setting. Given that its inclusion criteria also stated that “Patients who are candidates for and have access to anti-VEGF therapy (i.e., bevacizumab and regorafenib) and anti-EGFR therapy (i.e., cetuximab and panitumumab) and/or TAS-102 must have received appropriate therapy”,^[12]^ the guideline-recommended regimen should be raltitrexed or none (best supportive care). We apologize for this misclassification and corrected it throughout the revised manuscript.

Per the revised guideline-recommended regimen, the control group (GB201 [napabucasin]) of CTR20171559 [NCT03522649] was also deemed as suboptimal for two reasons. First, it was neither authorized by any regulatory approval agency before its ethical approval (December 2017, or even by now) nor recommended by any guideline for a specific indication. Second, although no standard chemotherapy was confirmed to be effective for refractory colorectal cancer patients for whom all available standard therapies had failed, the CO23 [NCT01830621] published before the start of CTR20171559 [NCT03522649] (April 2018) has demonstrated that overall survival did not differ significantly between GB201 [napabucasin] and placebo (4.4 vs 4.8 months, HR=1.13 95% CI 0.88-1.46, *p*=0.34).^[13]^ Results on comparative efficacy between GB201 [napabucasin] plus bi-weekly FOLFIRI and GB201 [napabucasin] could not inform future clinical decisions while attending this trial will result in an opportunity cost for cancer patients with short survival time.

Similarly, CTR20201983 was actually conducted in locally advanced esophageal cancer patients in the maintenance setting after CCRT, where "CCRT" is not the guideline-recommended regimen.

**Authors:** Thanks. We checked *Supporting Information* S1 Table 3 and found that “CCRT” should be the previously received regimen and was mis-filled in the “Guideline Recommended Regimen” column. We corrected this error and replaced it with “none” since the *Guideline of CSCO-Esophageal Cancer (2020 Edition)* declared that “there is no high-level evidence on the clinical benefit of consolidation chemotherapy after radical concurrent chemoradiotherapy”. Therefore, this trial was reclassified as “optimal control”.

It is also possible that the guidelines may not be updated timely to include the latest RCT results, leading to misclassified "suboptimal" control arms. For instance, CTR20212838 utilized SOX as the chemotherapy backbone in preoperative treatment for patients with locally advanced gastric cancer, which was supported by the positive results from the RESOLVE trial (doi: 10.1016/S1470-2045(21)00297-7) but not yet incorporated into the guideline at that time. As such, I would like to suggest the authors carefully re-assess the validity of the control arms of the included trials (if feasible, re-evaluation by oncologists in the field of specific cancer types).

**Authors:** Thank you for this comment. We fully agree that some guidelines may not be updated timely, as also declared by other research.^[14]^ We acknowledged this in the Limitation section. In Page 19 Line 397, we wrote:

*“Third, some guidelines may be subject to flaws, such that they were not updated timely or they expanded to include some options that were actually not relevant to the population being studied.[43]”*

To address this issue as much as possible, we checked whether the control regimen was deemed optimal in any revised guidelines later, and recategorized them as “potentially optimal control”. In Page 10 Line 186, we wrote:

*“For multinational trials, in consideration of the delay in new drug approval between China and other countries,[27] we further reviewed the Drugs@FDA database[28] to check whether the control regimen had been approved in the US, where most new cancer therapies were approved first,[29] before its date of IRB approval in China. If a multinational trial encompassed a specific indication on which a treatment consensus had not been reached in China, we checked whether there existed a standard of care in the former National Comprehensive Cancer Network (NCCN) Guidelines, which have a worldwide influence on clinical practice,[30] at that time. Moreover, given the time lag in updating clinical guidelines,[31] we also investigated whether a potentially suboptimal control regimen was deemed optimal in any revised guidelines later. Regimens that had been approved by the FDA, recommended as a standard-of-care in the NCCN guideline, or recommended in subsequent Chinese guidelines were recategorized as ‘potentially optimal control’.”*

As for the scenario (CTR20212838, the trial that investigated perioperative therapy for resectable gastric or gastro-esophageal junction adenocarcinoma) you kindly pointed out. We discussed this with the gastrointestinal oncologists. The RESOLVE study recruited adult patients with historically confirmed cT4a N+ M0 or cT4b Nany M0 gastric or gastro-esophageal junction adenocarcinoma (**Stage III**).^[15]^ Its results showed that adjuvant-SOX was non-inferior to adjuvant-CapOx in patients with locally advanced gastric cancer who had D2 gastrectomy in disease-free survival. Although median overall survival had not yet been reached at the time of the database lock, no statistically significant survival gain was observed between the two groups (HR=0.77, 95%CI 0.60-1.00). For Stage III gastric or gastro-esophageal junction adenocarcinoma, adjuvant SOX has been recommended in the 2021 CSCO Guideline-Gastric Cancer. For **Stage II** patients who were not eligible for the RESOLVE study, the S-1 single agent got a Grade I recommendation in 2021 and 2022 versions of specific guidelines while SOX got a Grade II recommendation.

CTR20212838 [NCT05149807] included adult patients who were “Pathologically diagnosed with GC or GEJC, histologically confirmed to be adenocarcinoma, and have no previous anti-tumor treatments for GC or GEJC” and “Be suitable for (investigator's assessment) and planning to undergo neoadjuvant therapy + radical surgery with curative intent before entering into the study”, which means that both Stage II and Stage III patients were eligible for participating in this trial. For Stage II patients, adjuvant-SOX was deemed as “suboptimal” since there existed higher-level recommendations.

We appreciated the Reviewer for pointing out the potential misclassification of our sample RCTs and checked all the results with our coauthoring oncologists. After careful review of the recommendations we used, in the revised manuscript, we reclassified two cases deemed as suboptimal in the previous criterion Category 4 (i.e., the control had a Grade I recommendation but there existed higher-level evidence in the same recommendation class. CTR20191073, CTR20190416). One other misclassification (CTR20170701) was also corrected in the revised results.

2.The authors reported that the proportion of trials using a suboptimal control arm was substantially higher in China-only trials than in international trials (21.3% vs 8.3%). One potential reason for this disparity is that the authors reviewed NCCN guidelines only for international trials but not China-only trials, while some of the "suboptimal" control arms in China-only trials may be supported by NCCN guidelines. Therefore, I recommend re-evaluating the validity of the control arms in China-only trials by considering both CSCO and NCCN guidelines as references.

**Authors:** Thank you for this comment. As was also pointed out by Reviewer #1, the credibility of Chinese clinical practical guidelines would affect the assessment of the appropriateness of a control arm. As stated by the CSCO Guideline Committee, the CSCO Guidelines were developed following the level of evidence and expert consensus. They are more relevant to clinical practices in China.^[16]^ Besides, the absence of an internationally harmonized standard of care, mainly driven by the unequal accessibility to some novel therapies, made it difficult to assess all RCTs registered in China against the US standard of care. Thus, we referred to Chinese clinical practical guidelines to assess the trials recruiting patients only from China. For multinational trials encompassed a specific indication on which a treatment consensus had not been reached in China, we further reviewed whether there existed a standard of care in the former National Comprehensive Cancer Network (NCCN) Guidelines, which have a worldwide influence on clinical practice, at that time (Page 10, Line 189).

Although these guidelines were developed under rigorous methodology, due to the absence of their methodology appraisal, they may have flaws. This was also clarified in the *Limitation* section. In Page 19, Line 397, we wrote:

*“Third, some guidelines may be subject to flaws, such that they were not updated timely or they expanded to include some options that were actually not relevant to the population being studied. In some CSCO Guidelines, treatments that are expensive but may have substantial benefits for the patients are also regarded as Grade II recommendations, which may bias the results on Category 3. Besides, although we used FDA approvals and NCCN recommendations to re-evaluate the quality of control arm of multinational trials, clinical benefits and level of evidence supporting their approval and recommendation may have discrepancies and deficiencies.[43, 44] To mitigate these impacts, we invited several oncologists and clinical pharmacists to cross-check if our results were in line with their observations in actual clinical practices.”*

In addition, previous literature, which was kindly provided by Reviewer#1, showed that only 13% of pivotal trials supporting FDA cancer drug approval recruited patients from upper-middle-income countries.^[17]^ The “trial recruitment bias” might also be a contributor to the discrepancies between China-only and international trials.

For that reason, we added this explanation in the *Discussion* section. In Page 17, Line 344, we wrote:

*“Compared with international multicenter RCTs, trials that recruited participants only in China seemed more likely to adopt an inferior control. A potential explanation is that trials initiated by multinational companies are more likely to recruit participants in high-income countries and thus the proportion of their trials using a suboptimal control might be underestimated.”*

3.The authors utilized the level of recommendation from the CSCO guidelines as one of the criteria for defining a suboptimal control arm. However, it is worth noting that CSCO guidelines' level of recommendation takes into account various factors, including clinical efficacy data and whether the regimens are included in the medical insurance reimbursement list. It should be acknowledged that the latter criterion might not be reasonable for defining the validity of control arms if a trial did not violate the former criterion, particularly since the primary endpoint for registration RCTs is focused on efficacy rather than reimbursement considerations.

**Authors:** Thank you very much for this thoughtful comment. We fully agree that therapy accessibility is also taken into consideration by CSCO Guidelines (“treatments that are expensive but may have substantial benefits for the patients are also regarded as Grade II recommendations”^[18]^). Therefore, we re-checked the affordability of the control group of the 21 trials in Category 3 (i.e., the comparator was recommended but there existed a higher-level recommendation). None of them was expensive on-patent medicine or not listed in China’s National Reimbursement Drug List, except for ARX788 versus lapatinib plus capecitabine for locally advanced or metastasis HER2-positive breast cancer patients who have progressed on trastuzumab (CTR20201708). A phase II study (NCT02422199), however, published before the IRB approval of CTR20201708 had demonstrated statistically significant better overall response rate and progression-free survival than lapatinib plus capecitabine.^[19]^ This makes lapatinib plus capecitabine classified as suboptimal control in Category 3. We also added this in the *Limitation* section as suggested. In Page 19, Line 397, we wrote:

*“Third, some guidelines may be subject to flaws, such that they were not updated timely or they expanded to include some options that were actually not relevant to the population being studied. In some CSCO Guidelines, treatments that are expensive but may have substantial benefits for the patients are also regarded as Grade II recommendations, which may bias the results on Category 3. …… To mitigate these impacts, we invited several oncologists and clinical pharmacists to cross-check if our results were in line with their observations in actual clinical practices.”*

4.The authors only included trials studying small-molecule entities and biologics. It would be helpful to clarify the reason for not including trials studying other drug types, such as chemotherapy.

**Authors:** Sorry for the confusion. The small-molecule entities included chemotherapeutic agents and molecule-targeted drugs. We further clarified this in the Method Section. In Page 9 Line 169, we wrote:

*“We included small-molecule entities (i.e., chemotherapeutic agents and molecule-targeted drugs) and biologics.”*

1. Zhang L, Li Y, Zhang C, Huang L, Liang Y, Han L. Analysis on status of clinical guidelines and evaluation on evidence-based guidelines of children in China. Zhongguo xun zheng yi xue za zhi. 2011;11:991-999.
2. Shah M, Rahman A, Theoret MR, Pazdur R. The Drug-Dosing Conundrum in Oncology - When Less Is More. N Engl J Med. 2021;385(16):1445-1447. doi:10.1056/NEJMp2109826
3. Oranratnachai S, Rattanasiri S, Pooprasert A, et al. Efficacy of First Line Systemic Chemotherapy and Multikinase Inhibitors in Advanced Hepatocellular Carcinoma: A Systematic Review and Network Meta-Analysis. Front Oncol. 2021;11:654020. Published 2021 Mar 31. doi:10.3389/fonc.2021.654020
4. Han Y, Zhi WH, Xu F, Zhang CB, Huang XQ, Luo JF. Selection of first-line systemic therapies for advanced hepatocellular carcinoma: A network meta-analysis of randomized controlled trials. World J Gastroenterol. 2021;27(19):2415-2433. doi:10.3748/wjg.v27.i19.2415
5. Woyach JA, Ruppert AS, Heerema NA, et al. Ibrutinib Regimens versus Chemoimmunotherapy in Older Patients with Untreated CLL. N Engl J Med. 2018;379(26):2517-2528. doi:10.1056/NEJMoa1812836
6. Wang FH, Zhang XT, Li YF, et al. The Chinese Society of Clinical Oncology (CSCO): Clinical guidelines for the diagnosis and treatment of gastric cancer, 2021. Cancer Commun (Lond). 2021;41(8):747-795. doi:10.1002/cac2.12193
7. Chen YP, Ismaila N, Chua MLK, et al. Chemotherapy in Combination With Radiotherapy for Definitive-Intent Treatment of Stage II-IVA Nasopharyngeal Carcinoma: CSCO and ASCO Guideline. J Clin Oncol. 2021;39(7):840-859. doi:10.1200/JCO.20.03237
8. Morris VK, Kennedy EB, Baxter NN, et al. Treatment of Metastatic Colorectal Cancer: ASCO Guideline. J Clin Oncol. 2023;41(3):678-700. doi:10.1200/JCO.22.01690
9. Oncology Society of Chinese Medical Association; Chinese Medical Association Publishing House. Zhonghua Zhong Liu Za Zhi. 2021;43(6):591-621. doi:10.3760/cma.j.cn112152-20210207-00118
10. Hilal T, Sonbol MB, Prasad V. Analysis of Control Arm Quality in Randomized Clinical Trials Leading to Anticancer Drug Approval by the US Food and Drug Administration [published correction appears in JAMA Oncol. 2019 Jun 20;:]. JAMA Oncol. 2019;5(6):887-892. doi:10.1001/jamaoncol.2019.0167
11. Mohyuddin GR, Koehn K, Sborov D, et al. Quality of control groups in randomised trials of multiple myeloma enrolling in the USA: a systematic review. Lancet Haematol. 2021;8(4):e299-e304. doi:10.1016/S2352-3026(21)00024-7
12. https://clinicaltrials.gov/study/NCT03522649?id=NCT03522649&rank=1
13. Jonker DJ, Nott L, Yoshino T, et al. Napabucasin versus placebo in refractory advanced colorectal cancer: a randomised phase 3 trial. Lancet Gastroenterol Hepatol. 2018;3(4):263-270. doi:10.1016/S2468-1253(18)30009-8
14. Bouche G, Meheus L. Ensuring completeness and timeliness of cancer treatment guidelines. Nat Rev Clin Oncol. 2022;19(9):563-564. doi:10.1038/s41571-022-00646-w
15. Zhang X, Liang H, Li Z, et al. Perioperative or postoperative adjuvant oxaliplatin with S-1 versus adjuvant oxaliplatin with capecitabine in patients with locally advanced gastric or gastro-oesophageal junction adenocarcinoma undergoing D2 gastrectomy (RESOLVE): an open-label, superiority and non-inferiority, phase 3 randomised controlled trial [published correction appears in Lancet Oncol. 2021 Aug;22(8):e347]. Lancet Oncol. 2021;22(8):1081-1092. doi:10.1016/S1470-2045(21)00297-7
16. Li Q, Liu J, Jiang Z, Liu Q. CSCO breast cancer guideline: precise, economical and oriental. Sci China Life Sci. 2020;63(9):1410-1412. doi:10.1007/s11427-020-1701-5
17. Awan FA, Becker AB, Wang Y, Kimmelman J. Participant Recruitment From Low- and Middle-Income Countries for Pivotal Trials of Drugs Approved by the U.S. Food and Drug Administration : A Cross-Sectional Analysis. Ann Intern Med. 2022;175(12):1675-1684. doi:10.7326/M22-1857
18. Wang FH, Zhang XT, Li YF, et al. The Chinese Society of Clinical Oncology (CSCO): Clinical guidelines for the diagnosis and treatment of gastric cancer, 2021. Cancer Commun (Lond). 2021;41(8):747-795. doi:10.1002/cac2.12193
19. Ma F, Ouyang Q, Li W, et al. Pyrotinib or Lapatinib Combined With Capecitabine in HER2-Positive Metastatic Breast Cancer With Prior Taxanes, Anthracyclines, and/or Trastuzumab: A Randomized, Phase II Study. J Clin Oncol. 2019;37(29):2610-2619. doi:10.1200/JCO.19.00108
