# Supplementary material for: Use of suboptimal control arms in randomized clinical trials of investigational cancer drugs in China, 2016–2021: An observational study
Source: PLoS Med. 2023 Dec 12;20(12):e1004319. doi: 10.1371/journal.pmed.1004319 (PMC10715645; doi:10.1371/journal.pmed.1004319)
Supplement: Attachment — Submitted filename: 1_Author Response.docx [file pmed.1004319.s004.docx]

**COMMENTS FROM THE EDITORS**

**GENERAL**

Thank you for your very detailed and considered responses to previous editor and reviewer comments. Please see below for further comments which we require that you address prior to publication.

The reviewer (please see below) notes that you report 84% and 100% of positive primary outcomes in RCTs which we agree is very high. We agree with the reviewer that this warrants further discussion as part of further revisions.

**Authors:** Thank you and Reviewer#1 for this comment. We gave this issue a second thought. One of the potential explanations is that our sample included randomized oncology registration trials in 2016-2021, which may have not been completed by the end of our observation. With a median follow-up period of 3.4 years following IRB approval, only 23.2% of included trials have publicly available results (Page 14 Line 276). The selective reporting of results, gearing towards those positive, maybe another reason. We further discussed this finding in the revised *Discussion* section as suggested. In Page 16 Line 330, we wrote:

*“[N]ot comparing an investigational regimen against a proven best therapy generates suboptimal evidence. Our results also showed that, compared with trials using optimal control, those adopting a suboptimal control showed a higher proportion of concluding positive results, which favored the pharmaceutical companies, though this was not statistically significant, and only 23.2% of sample trial results were publicly available with limited follow-up time. It was noteworthy that 83.9% (78/93) and 100.0% (12/12) of our sample RCTs with optimal and suboptimal control concluded positive primary endpoint(s) respectively, which was higher than those (51.4% [177/344] and 76.7% [33/43] of solid tumor trials published among 11 major oncology journals) reported in a recent literature [11]. Given that this study focused on randomized oncology registration trials in China, one of the concerns is the selective reporting of results, geared towards those positive, may cause patients and regulators to exaggerate the clinical benefits of the investigational drugs. If this practice is widespread in the development pipeline, it may hamper therapeutic innovation and threaten the promotion of optimal clinical practice in the long term [8].”*

Additionally, the high proportion of RCTs reporting positive results may also be attributed to publication bias. This was also clarified in the *Limitation* section. In Page 20 Line 411, we wrote:

*“Sixth, publicly available data may represent a selective subset of trial information since only 23.2% of sample trial results were published by the end of our observation.”*

In line with the above the editorial team agree that discussion of how these data compare to the same/similar outside of China for cancer drugs and inside of China for other specialty meds (e.g Rheumatology) would be of value to better ascertain if this a country-specific issue or a specialty-specific issue – i.e. specific to cancer drugs. The Academic Editor also agrees, please see below.

**Authors:** Thank you very much for this insightful advice. In the *Discussion* section, we compared our findings with other countries’ research on cancer drugs. In Page 16 Line 312, we wrote:

*“[E]vidence showed that 17-25% of superiority RCTs supporting FDA approval of cancer drugs in 2013-2019 used a substandard control arm [5, 33]. Similarly, we found that approximately one-eighth of RCTs for new cancer drug applications in China between 2016 and 2021 were conducted using a suboptimal comparator. Adoption of a control regimen known to be inferior to available therapies or not recommended by specific guidelines was the primary contributor, as found in this research and in the literature [5]. Although it is challenging to compare regulatory outcomes across different settings and time periods, it seems that the proportion of RCTs registered in China with an optimal control was comparable to those in other developed countries, which suggests that trials registered in China are no less aligned with contemporary ethical standards than are in the U.S. or European countries.”*

To the best of our knowledge, there is no study on the quality of control arms of other specialty medicine trials in China. Previous international literature, however, has talked about the absence of head-to-head trials and control arms among biologic treatment of rheumatoid arthritis and direct oral anticoagulants. We therefore added a discussion on the selection of control for these diseases. In Page 16 Line 322, we wrote:

*“Adoption of a suboptimal control in RCTs is also prevalent in various diseases apart from oncology, such as rheumatology and cardiology [35, 36]. It could be explained from the perspectives of study investigators, regulators, and the landscape change of cancer treatment.”*

**COMMENTS FROM THE ACADEMIC EDITOR**

Agree; the authors hint at the international comparison issue in the intro, but don't mention it again. Since this study is so laser-focused on China, would be useful to explain why they're focusing on China drug policy and how the Chinese FDA works/regulates in this area, at least as compared to FDA and EMA. Are these studies new studies done expressly for Chinese FDA approval, or are they the same pivotal studies submitted to other international regulators?

**Authors:** Thank you for this helpful comment. We chose to focus on China’s policy as China issued the *Guidance on Clinical Value-Oriented Oncology Drug Research and Development* in November 2021, aiming to promote a better generation of clinically relevant novel oncology drugs in China. Although the guidance suggests that an active control arm should be selected with considerations of whether it reflects the optimal treatment in current clinical practices, its definition of “optimal treatment” is ambiguous. This was also emphasized in the *Author Summary* (Page 4 Line 65) and *Discussion* (Page 28, Line 356) section. We hope our research findings can provide empirical evidence to the stakeholders and draw regulators’ attention to this matter so that the guideline can be delivered in the manner that it set out to be.

For the latter comment, all of our sample RCTs were registered with the intent to attain regulatory approval by China’s National Medical Products Administration. We further checked whether they were the same pivotal trials supporting relevant FDA cancer drug approvals in 2000-2020, using databases we built previously.^[1]^ ^[2]^ The crude results showed that only 2.2% (10/453, NCT03069352, NCT02993523, NCT02775435, NCT02409342, NCT02477826, NCT03043872, NCT03215706, NCT02489318, NCT02987543, NCT03085095) of them were the same trials.

Line 26 – please remove the competing interest statement and include only in the manuscript submission form when you re-submit the manuscript. It will be compiled as metadata at the time of publication.

**Authors:** We removed the *Competing Interest* statement from the manuscript as required.

**AUTHOR SUMMARY**

Thank you for including an author summary.

Line 81 - What Did the Researchers Do and Find? Do you mean upward trend?

**Authors:** Sorry for the missing word. We corrected this sentence following your kind comment. In Page 5 Line 74, we wrote:

*“We observed a fluctuating yet overall upward trend in the number of trials and participants that used suboptimal comparators during our observation period.”*

Line 90 – What Do These Findings Mean? Please make sentence beginning ‘Regulatory agencies…’ a separate bullet point.

**Authors:** Thanks. We split this sentence into separate bullet points as suggested. In Page 5 Line 81, we wrote:

- *“Trial sponsors, ethical review boards, and oncologists should make collaborative efforts to protect patients from unnecessary harm and drugs with uncertain clinical benefits over the existing standard of care.*
- *Regulatory agencies should be cautious when reviewing investigational new drug applications whose supporting trial used a suboptimal control and when making subsequent market authorization decisions.”*

**REFERENCES**

For in-text reference callouts citations should be placed in square brackets preceding punctuation. For example, line 100 should read, ‘for evaluating new drugs [1, 2].’ Please also note the addition of a space between the text and opening bracket. Please check an amend throughout all sub-section of manuscript.

**Authors:** We placed citations preceding punctuation and added a space between the text and the opening bracket throughout the manuscript as required.

**SUPPORTING INFORMATION**

Thank you for referencing and reporting yours according to PRISMA considering the search strategy applied to collection and extraction of your data. For completeness, please also include the PRISMA checklist as supporting as information. When completing the checklist, please use section and paragraph numbers, rather than page numbers.

Please add the following statement, or similar, to the Methods: "This study is reported as per the Preferred Reporting Items for Systematic Reviews and Meta-Analyses (PRISMA) guideline (S1 Checklist)."

**Authors:** Thanks. We filled in the PRISMA checklist using section and paragraph numbers and uploaded it as supporting information. In Page 8 Line 153, we wrote:

*“The review adhered to the Preferred Reporting Items for Systematic Reviews and Meta-Analyses (PRISMA) guideline (see S2 Checklist).”*

**COMMENTS FROM REVIEWERS**

**Reviewer #1:** This paper continues to be a valuable contribution to the meta-research literature on randomized trials. It is well done, and address an important gap in the literature. The revisions address all of my concerns. There is one last point I want to raise- the authors may want to address it.

In revisions, the authors find that 84% and 100% of RCTs produced positive outcomes on their primary. That is just plain weird, and implies a lack of equipoise in the trials (on average, RCTs are positive 30-45% of the time; this reflects that answers of trials should be unknowable in advance of their conduct- see work of Benjamin Djulbegovic). To me, this high positive rate raises some concerns - perhaps orthogonal to the subject of the manuscript- about design or reporting practices used for this trial sample. I encourage authors- if they agree- to perhaps add a sentence or two in the discussion about this odd result.

**Authors:** Thank you for this perceptive comment. We further interpreted this finding in the revised *Discussion* section, as suggested by you and the Editor. In Page 16 Line 330, we wrote:

*“[N]ot comparing an investigational regimen against a proven best therapy generates suboptimal evidence. Our results also showed that, compared with trials using optimal control, those adopting a suboptimal control showed a higher proportion of concluding positive results, which favored the pharmaceutical companies, though this was not statistically significant, and only 23.2% of sample trial results were publicly available with limited follow-up time. It was noteworthy that 83.9% (78/93) and 100.0% (12/12) of our sample RCTs with optimal and suboptimal control concluded positive primary endpoint(s) respectively, which was higher than those (51.4% [177/344] and 76.7% [33/43] of solid tumor trials published among 11 major oncology journals) reported in a recent literature [11]. Given that this study focused on randomized oncology registration trials in China, one of the concerns is the selective reporting of results, geared towards those positive, may cause patients and regulators to exaggerate the clinical benefits of the investigational drugs. If this practice is widespread in the development pipeline, it may hamper therapeutic innovation and threaten the promotion of optimal clinical practice in the long term [8].”*

Additionally, the high proportion of RCTs reporting positive results may also be attributed to publication bias. This was also clarified in the *Limitation* section. In Page 20 Line 411, we wrote:

*“Sixth, publicly available data may represent a selective subset of trial information since only 23.2% of sample trial results were published by the end of our observation.”*

**Reviewer #2:** Thank you to the authors for addressing my previous comments well. I have no further issues to raise.

**Reviewer #3:** I appreciate the authors' efforts on sufficiently addressing my concerns. I have no further comments.

**Authors:** We thank the Reviewers for their valuable comments.

1. Naci H, Guan X, Woloshin S, Xu Z, Wagner AK. Communication of Survival Data in US Food and Drug Administration-Approved Labeling of Cancer Drugs. JAMA Intern Med. 2021;181(11):1521-1522. doi:10.1001/jamainternmed.2021.3505
2. Zhang Y, Katharina Wagner A, Du H, et al. Childhood cancer drugs in China: An overview and comparison of regulatory approvals in China and the United States. Int J Cancer. 2022;150(3):482-490. doi:10.1002/ijc.33818
